# Temperature extremes contribute to suicide-related help-seeking through multiple pathways: Evidence from crisis hotline data (2019–2023)

**DOI:** 10.1371/journal.pmen.0000501

**Published:** 2026-02-11

**Authors:** Sophia C. Ryan, Margaret M. Sugg, Victoria Schwandt, Sherrard Crespo, Summer Lindzey, Jennifer D. Runkle

**Affiliations:** 1 Department of Geography and Environment, University of North Carolina at Chapel Hill, Chapel Hill, North Carolina, United States of America; 2 Department of Geography and Planning, Appalachian State University, Boone, North Carolina, United States of America; 3 VIA LINK, Covington, Louisiana, United States of America; 4 North Carolina Institute for Climate Studies, North Carolina State University, Asheville, North Carolina, United States of America; National Psychological Association of Ukraine, UKRAINE

## Abstract

This study investigated how nighttime temperatures influence suicidal help-seeking behavior via theorized pathways: sleep disruption, impulsivity, social isolation, and unmet basic needs. We analyzed 11,684 suicide hotline risk assessments (Louisiana, USA). Distributed lag nonlinear models quantified temperature associations with standardized suicide risk factors during minimum temperature extremes (≤10th, ≥ 90th percentiles). Natural language processing on counselor notes identified help-seeking crisis contexts; generalized additive models examine associations between crisis contexts and temperature exposure. Stratified demographic analyses provide exploratory insights into mechanistic pathways across populations. Suicide-related help-seeking increased dramatically with rising nighttime temperatures: 19% at the 90th percentile (PR = 1.19, 95% CI: 1.10-1.29), 55% at the 95th percentile (PR = 1.55, 95% CI: 1.44-1.68), and 166% at the 99th percentile (PR = 2.66, 95% CI: 2.42-2.92); an estimated absolute increase of 19 suicide calls per 100 crisis calls in the two days following hot nights. These increases were accompanied by sleep difficulties (+146% at 99th percentile), expressed intent to die (+163% at 99th percentile), availability of lethal means (+145% at 99th percentile), and few future plans (+224% at 99th percentile). Crises related to mental health (+20.9%) and basic needs (+59.9%) increased at the 90th percentile; sleep was more prevalent at cold temperatures (+68.1% at 10th; -31.9% at 90th). Youth, women, and Black clients were associated with mental health mentions at high temperatures; youth were also associated with increases in isolation. Black clients and men mentioned substance use more during high temperatures. Temperature extremes exacerbate suicide risk pathways, notably sleep disruption, substance use and impulsivity, essential needs stress, and social isolation. Results are most applicable to other humid subtropical climates with similar sociodemographics and should be interpreted as exploratory due to high demographic missingness. Findings inform proactive interventions, such as temperature-triggered staffing protocols and integration of basic needs support into suicide prevention during extreme weather events.

## 1. Introduction

The relationship between ambient temperature and suicide risk is one of the most consistent findings in environmental health research. Extensive literature has identified a strong link between high temperatures and increased risk of suicidality [1–4], with estimates suggesting that temperature can explain between 15% [5] and 60% [6] of the variability in suicide rates worldwide. Prior work highlights that the effects of temperature extremes on suicidality differ among youth, women [7,8], older adults [5], and Hispanic individuals [7], who are particularly vulnerable. However, the reliance of this literature on mortality data [2] can obscure the mechanisms connecting temperature exposure to suicide risk. Understanding crisis help-seeking patterns and precipitating stressors during temperature extremes can inform how and when to intervene, thereby reducing barriers to translating epidemiological evidence into effective prevention practices.

Four primary theoretical pathways can help understand the relationship between temperature and suicidality. Two pathways are behavioral in nature: the temperature-aggression hypothesis [9,10] and the routine activity theory [11], the third is physiological, driven by poor sleep [12], and the fourth, the motivation-volitional theory, is driven by entrapment and defeat [13], which are exacerbated by economic and resource stress [14]. The temperature-aggression hypothesis posits that heat physiologically increases impulsivity [15,16], aggression [15], violence [17,18] and self-harm [19]. Indeed, prior work has found that interpersonal violence [20], impulsivity [16], and abuse against women [21] are higher during periods of hot ambient temperatures. Another behavioral pathway, the routine activity theory, posits that extreme temperatures change an individual’s routine, potentially increasing opportunities for interpersonal conflicts and crime [11], with social isolation identified as a particularly important temperature-related risk factor among older adults [22]. Sleep disruption and cognitive impairment, physiological pathways which increase during extreme heat, mediate the temperature-suicide relationship, contributing to increased incidence of suicide deaths and attempts [12]. Reports of poor sleep and reduced cognitive functioning are more common during high temperatures, offering evidence to this pathway [23–26]. Finally, the integrated motivation-volitional theory offers a lens to understanding that extreme temperature events can trigger cascading disruptions in access to basic resources, compounding psychological distress among already vulnerable populations [14]. The integrated motivational–volitional theoretical model of suicide offers a useful framework for understanding this relationship suggesting that basic needs stressors, such as energy poverty and economic hardship, can generate a sense of entrapment and hopelessness that escalates suicidal ideation and behavior [13,14].

Despite supporting evidence, retrospective mortality and morbidity studies are often unable to measure the upstream mechanisms that drive temperature-related crises, thereby hindering the development of targeted prevention strategies. Furthermore, while a large body of research has linked high temperatures with increased suicidality, emerging evidence highlights that extreme cold weather events (e.g., ice storms) are significantly associated with long-term increases in suicide-related burdens [27] and depression [28]; this work stresses the need for a more critical lens into extreme temperatures and suicide. Additional empirical evidence regarding precipitating concerns and help-seeking behaviors in the context of temperature extremes, both hot and cold, is needed to fill a critical gap in climate-suicide research with urgent implications for policy and prevention.

Despite robust evidence linking high temperatures to suicide risk, the co-occurring stressors and precipitating contexts remain largely unexamined. We address this gap by analyzing real-time crisis hotline data from Louisiana, a state experiencing both extreme heat (humid subtropical climate) and extreme cold events (e.g., Severe Winter Storm Uri in 2021), to identify not only when but why people seek suicide-related help during temperature extremes. Using natural language processing on counselor narratives, coupled with distributed lag nonlinear models, we test whether specific stressors align with theoretical pathways: sleep disruption (physiological pathway), aggression and impulsivity (temperature-aggression pathway), isolation and interpersonal conflict (routine activity pathway), and basic needs stress (motivational-volitional pathway). We hypothesize that these mechanisms will manifest differently across hot versus cold extremes and demographic groups (age, gender, race), with thematic analyses revealing additional context on population-specific vulnerabilities. By capturing crisis help-seeking patterns before potential suicide attempts, this approach helps reveal actionable intervention targets invisible in mortality data.

## 2. Methods

### 2.1 Study area

Louisiana, located in the southern United States, is home to an estimated 4.5 million people [29]. Sociodemographically, the population is estimated to be 56.6% non-Hispanic white, 32.6% non-Hispanic Black, and 7.3% Hispanic; approximately 18.9% of the population lives in poverty, and an estimated 85% of households have broadband internet access [29]. Characterized by a humid subtropical climate, winters are relatively mild and short-lived, and summers are hot [30]. While daytime temperatures in Louisiana have not increased substantially in the recent climate record (i.e., mid-20th century - present), minimum temperatures, a proxy for nighttime temperatures, have risen consistently since 2000 [30]. Louisiana’s climate is partially influenced by its proximity to the Atlantic Gulf Coast; precipitation occurs throughout the year, and hurricanes are a semi-regular occurrence, with an average of one hurricane hitting the state every three years [30].

**Fig 1:**
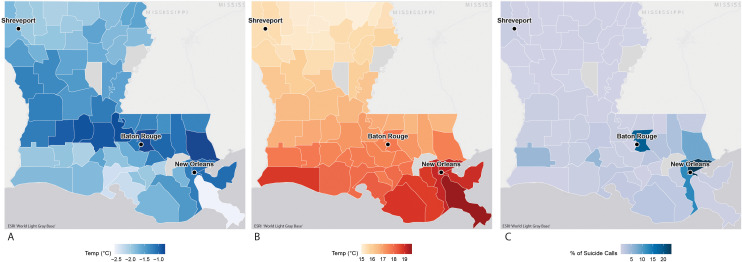
Panel map of Louisiana depicting (A) median minimum temperatures at the 10th percentile, (B) median minimum temperatures at the 90th percentile, and (C) percent of suicide crisis calls by county. The three largest urban areas are labeled (New Orleans, Baton Rouge, Shreveport). Temperature data was obtained from PRISM; suicide conversation data from VIA LINK (2019-2023). ‘Map image is the intellectual property of Esri and is used herein under license. Copyright © 2025 Esri and its licensors. All rights reserved. Basemap is ESRI World Light Gray Base (https://www.arcgis.com/home/item.html?id=ed712cb1db3e4bae9e85329040fb9a49).’.

The coldest minimum temperatures ≤10th percentile were observed in central Louisiana, encompassing the Baton Rouge metropolitan area, while the hottest minimum temperatures ≥90th percentile were observed in the New Orleans metropolitan area (Fig 1). The percentage of crisis conversations is highest in southeast Louisiana, particularly in Baton Rouge and New Orleans, corresponding to the primary service area of VIA LINK [31].

### 2.2 Suicide help-seeking data

Suicide-related crisis data were obtained from VIA LINK, a non-profit crisis organization offering free 24/7 crisis and information services, operating numerous lines in the region, including national hotlines like 988 and 211 [31]. Individuals in crisis can call, chat, or text to be paired with trained crisis counselors, who provide emotional support, referrals, information, and crisis counseling. VIA LINK is the primary operator for 988 in the state, contracting with the National Crisis Hotline to provide free crisis services to clients in Louisiana. VIA LINK also operates the Teen Crisis Textline, a targeted crisis and mental health hotline for youth in Louisiana, can be reached by dialing 833-TXT-TEEN (https://vialink.org/teen-text-program/) [31]. When VIA LINK clients exhibit suicide-related behaviors or share that they have experienced suicidal thoughts within the last 24 hours, crisis counselors are required to conduct standardized suicide risk assessments (SRAs).

Anonymized crisis hotline data was obtained through a data use agreement and includes narrative statements from the crisis counselors (i.e., conversation notes), providing contextual information on caller-identified suicide-related concerns. Counselor-collected demographic information (e.g., age, gender and race and ethnicity) is available for roughly 35% of conversations [age (available for ~44% of conversations), race and ethnicity (available for ~20% of conversations), and gender (available for ~65% of conversations)]; counselors also collect geographic information (e.g., county of residence), enabling place-based analysis. Data for this analysis consists of individuals who contacted crisis hotlines operated by VIA LINK and qualified for a SRA (i.e., active suicide ideation).

#### 2.2.1 Clinician-assessed suicide risk.

We analyzed crisis call data from 2019 to 2023 involving a SRA, yielding a sample of 11,684 cases. For each call, we derived binary indicators for five counselor-coded risk factors required for assessment during the SRA: decreased sleep, expressed intent to die, access to lethal means, few future plans, and difficulty engaging with counselors. These suicidal risk factors serve as proxies for established suicide risk correlates such as impulsivity and physiological dysregulation (Joiner et al., 2007). We opted for binary indicators rather than continuous measures to facilitate the construction of a daily, county-level time series representing the number of crisis calls per county per day.

#### 2.2.2 Conversation-derived help-seeking themes.

As a secondary indicator for suicidal mechanisms, we used an exploratory approach leveraging natural language processing to identify pre-specified crisis concern themes within conversation notes as a proxy for help-seeking context, including sleep, mental health, substance use, isolation, interpersonal stressors, and basic needs (i.e., housing, air conditioning, and utilities) (see S1 Table). Counselor call reports (i.e., narrative transcripts) were tokenized, lower-cased, and stemmed [32]. Custom keyword dictionaries were developed for each theme based on manual review of a random subset of 500 narratives and established associations with both suicide risk and extreme weather exposure, such as sleep disruption [33], isolation [22], and interpersonal stress [20]. Conversations were classified as mentioning a theme if they contained at least one keyword. Theme counts were aggregated to the daily level by county.

To establish inter-rater reliability, a coder manually coded a subset of 100 random narratives and compared manual coding to NLP-derived themes using Cohen’s kappa coefficient (*k* ranges 0–1). Overall, the inter-rater reliability (*k)* between manual and NLP-derived coding was: 0.95. No discrepancy was observed for basic needs, mental health, interpersonal and substance use (k: 1.0). False positives were present for sleep (k: 0.72) attributed to keyword detection out of context (e.g., tired of school) and for isolation (k: 0.72) attributed to ‘disconnect’ and ‘disconnected’ in the context of call connection (e.g., call disconnected). As an additional check, a subset of 50 narratives was manually coded by two independent coders, the overall *k* was 0.90 [basic needs: 1.0; mental health: 1.0; isolation: 0.70; substance use: 1.0; sleep: 0.70; interpersonal: 1.0]. As with the prior check, false positives were present for sleep (k: 0.70) attributed to keyword detection out of context (e.g., tired of school) and for isolation (k: 0.70) attributed to ‘disconnect’ and ‘disconnected’ in the context of call connection (e.g., call disconnected), which were treated differently between the two coders. Thematic identification utilized R packages, including tidytext, stringr, and custom keyword dictionaries, which were validated against a manual coding of a subset of conversations [34–36].

For this work, *clinician-assessed* refers to standardized suicide risk measures assessed by crisis counselors; *help-seeking themes* refers to NLP-derived context from crisis narratives. Mechanistic pathways (i.e., sleep disruption, aggression/impulsivity, interpersonal stress, and essential needs stress) are empirically tested using both the clinician assessed risk factors and help-seeking themes.

#### 2.2.3 Mechanistic pathways.

To empirically examine the four proposed mechanistic pathways, we leverage both clinician-assessed risk factors and NLP-derived help-seeking themes [1]. The temperature-aggression pathway was assessed using clinician-assessed indicators of impulsivity, including few future plans and expressed intent to die and NLP-derived substance use theme [2,15,16]. The routine activity theory was captured through difficulty engaging with crisis counselors (clinician-assessed) and NLP-derived help-seeking themes of isolation and interpersonal stress [3,11]. Physiological disruption to sleep was measured via clinician-assessed poor sleep and the NLP-identified help-seeking theme of sleep [4,12]. Finally, the motivation-volitional theory was assessed using the NLP-derived help-seeking theme of unmet basic needs and clinician-assessed access to lethal means [13,14].

### 2.3 Temperature data

Daily temperature data were obtained at 4 km grid intervals from PRISM at Oregon State [37]. Data was aggregated to the county level, with minimum temperatures derived by county as a daily time-series over five years, covering the period from January 1, 2019 to December 31, 2023. We prioritized minimum temperature as the primary exposure because it better represents nighttime conditions when sleep disruption occurs and has shown stronger associations with human health outcomes than daytime maximum [38]. Sensitivity analyses were also conducted for maximum and average temperature, common temperature measures in the suicide literature [2].

Temperature extremes were defined using percentile thresholds rather than absolute values to account for local adaptation and climate variability [39]. This relative approach has been validated in multi-country studies of temperature-mortality relationships, which found that populations exhibit adaptation to their local climate, making percentile-based thresholds more epidemiologically meaningful than fixed absolute values [4]. Hot days were identified as those with a minimum temperature at or above the 90th, 95th, and 99th percentiles. Cold days were defined as ≤10th, ≤ 5th, and ≤1st percentiles of minimum temperature.

### 2.4 Covariates

Our time period of 2019-23 encompasses the COVID-19 pandemic and multiple major hurricanes in Louisiana (e.g., Laura (2020), Ida (2021)). We adjust for these events using binary indicators (1/0). The pandemic is defined as the period from March 11, 2020, to May 11, 2023, aligning with the United States Public Health Emergency declaration [40]. Tropical cyclones exposures were assigned to county centroids within 50 miles of a tropical storm track on that day using the National Oceanic and Atmospheric Administration HURDAT storm track [41] data. Additional model covariates included year, day of week, and precipitation.

Individual-level covariates were considered in exploratory stratified analyses for age (young people aged 24 and under, and adults aged 25 and over), race (Black and White), and gender (male and female).

### 2.5 Statistical analysis

#### 2.5.1 Distributed lag nonlinear models - Suicide risk.

A small-area distributed lag nonlinear model (DLNM) was employed to model the lagged association between daily county minimum temperatures and suicide crisis calls, as well as counselor-coded risk factors. The small area approach is an extension of the DLNM, which allows for inclusion of geographic areas with small populations (e.g., rural communities) [42]. The DLNM approach and related extensions have been applied in prior temperature-mental health work [43–45].

This analysis employed a quasi-Poisson distribution to estimate the prevalence ratio (PR) of suicide-related service calls and accompanying risk factors (e.g., poor sleep, intent to die) on the day of and up to two days following days at or above the 90th, 95th and 99th minimum temperature percentiles (high temperatures) and at or below the 10th, 5th and 1st temperature percentiles (cold temperatures) [4].

DLNM models include a cross-basis function, which is a matrix that considers both exposure (temperature) and outcome (suicide-related help-seeking) for each lag period (i.e., 0–2 days). The cross-basis was created for each county using a natural spline with 3 knots placed at the 25th, 50th, and 75th temperature percentiles following the established literature (Y. Kim et al., 2019; Wu et al., 2024). The cross-basis matrix becomes the main predictor value in the generalized linear model, which adjusts for year, day of week, and precipitation (Y. Kim et al., 2019; Ulrich et al., 2025) The COVID-19 pandemic and exposure to tropical cyclones were accounted for using binary variables.

Results are reported as prevalence ratios (PR), calculated as the prevalence (or risk) of suicide crisis calls at temperature extremes (i.e., 90th, 95th, 99th, 10th, 50th, 1st) compared to the median temperature. Absolute risk difference (ARD) estimates contextualize results, reporting the predicted increase or decrease in call volume corresponding to the prevalence ratio estimates. ARD was calculated using ARD: α × (PR-1)/ PR, where PR represents the prevalence (risk) ratio for suicide risk where α reflects the baseline rate of HRI at the 50th percentile, scaled per 100 individuals (i.e., [SRA count at 50th/ total SRA count] × 100). Significance was derived at ɑ < 0.05. All analyses were conducted in R using the dlmn package [35,46].

#### 2.5.2 Generalized additive models - Help-seeking crisis concerns.

Daily temperature effects on help-seeking themes were examined using generalized additive models (GAMs) with penalized smoothing splines (k = 8). GAMs, as with DLNMs, model lagged and additive effects. We elected to use GAM models for help-seeking crisis concern theme analysis, as they are better equipped to handle smaller sample sizes compared to DLNMs [47,48], and several temperature-needs associations exhibited linear rather than non-linear associations. Following the modelling parameters used in the DLNM, we apply a two-day lag structure and model minimum temperature. The models adjust for day of the week and year to account for seasonal and weekly trends in help-seeking. The effective degrees of freedom (EDF) indicate the complexity of the temperature-theme association, with EDF = 1 representing linear effects and higher values indicating non-linear patterns. The minimum ambient temperature was used as the primary predictor, with significance derived at the 10th and 90th temperature percentiles at α < 0.05. GAMs were run in R using the ‘mgcv’ package [35,49].

### 2.6 Sensitivity analyses

We also conducted DLNM analyses using average and maximum temperatures, and an extended lag structure (0-10 days, e.g., [50]) to capture the full spectrum of thermal exposures and potential delayed effects in suicide-related help-seeking. While minimum temperatures are relevant for sleep disruption and nighttime vulnerability, average and maximum temperatures may influence different behavioral and physiological pathways, such as daytime exposure, activity patterns, and cumulative heat stress.

Additional sensitivities were stratified by demographics [age (available for ~44% of conversations) (youth ≤24 and adults ≥ 25 years), race (available for ~20% of conversations) (White and Black/African American), and gender (available for ~65% of conversations) (men and women)]. Despite the reduced sample sizes in some subgroups, this demographic stratification provides important exploratory insights into differential heat-related mental health impacts.

Additionally, we included a moderation analysis in our GAMs as a sensitivity, where we tested moderation by sleep, including a sleep × mental health interaction term in a GAM with the same model parameters as above. The significance of the interaction was tested with an ANOVA (α < 0.05). Stratified analyses compare interaction between sleep and mental health at temperature extremes for cold (<10th percentile), normal (10–90th percentiles), and hot (>90th percentile) minimum temperatures. We also conducted GAM analyses stratified by demographics (age groups: ≤ 24, ≥ 25; gender: men, women; race/ethnicity: white, Black) using the same parameters as our DLNM analysis.

## 3. Results

### 3.1 Descriptive statistics

Among 11,684 suicide risk assessments (2019–2023), demographic data were available for 21–66% of calls. The sample was predominantly adults ≥25 years (52.8%), white (52.2%), and women (55.9%) (Table 1). These demographics are similar to that of Louisiana: population ages 25 and over (~68%), white (56.6%) and women (51.0%), though our sample has higher representation of youth (47.2% in our sample vs 32.7% in Louisiana), Black help-seekers (40% in our sample vs 30.8% in Louisiana), and women (55.9% in our sample vs 51% in Louisiana), compared to the general Louisiana population (US Census, 2020).

**Table 1:** Demographics summary of suicide-related crisis conversations for the entire sample, and those above the 90th and below the 10th percentiles of minimum temperature.

Variable	Overall (%)	90th Percentile	10th Percentile
	11,684	1,236	1,168
**Age (44.1% of sample)**			
Youth 24 and Under	2426 (47.2)	240 (45.9)	229 (45.6)
Adults 25 and Over	2723 (52.8)	283 (54.1)	273 (54.4)
**Race and Ethnicity (21.7% of sample)**			
Black/ African American	1018 (40.0)	100 (36.9)	106 (40.5)
Hispanic/ Latino	80 (3.1)	7 (2.6)	7 (2.7)
Other and Mixed Race	114 (4.7)	12 (4.5)	10 (3.8)
White	1328 (52.2)	152 (56.1)	139 (53.1)
**Gender (65.6% of sample)**			
Female/Woman	4285 (55.9)	387 (52.2)	498 (61.2)
Male/Man	3281 (42.8)	350 (47.2)	309 (38.0)
TGD	99 (1.3)	4 (0.5)	7 (0.9)
Clinician Assessed Suicide Risk Factors* (100% of sample)			
Availability of Means	3165 (27.1)	324 (26.2)	353 (30.2)
Difficulty Sleeping	5019 (43.0)	531 (43.0)	507 (43.4)
Preparatory Behaviors	2163 (18.5)	206 (16.7)	242 (20.7)
Expressed Intent to Die	6661 (57.0)	716 (57.9)	649 (55.6)
Little Engagement with Crisis Counselor	2537 (21.7)	283 (22.9)	245 (21.0)
Few Future Plans	3657 (31.3)	423 (34.2)	380 (32.5)
**Help-Seeking Crisis Concerns+ (100% of sample)**			
Basic Needs	735 (6.3)	71 (5.7)	85 (7.3)
Mental Health	3158 (27.0)	317 (25.6)	346 (29.6)
Substance	308 (2.6)	30 (2.4)	35 (3.0)
Interpersonal	4662 (39.9)	481 (38.9)	485 (41.5)
Sleep	501 (4.3)	54 (4.4)	59 (5.1)
Isolation	1257 (10.8)	94 (7.6)	140 (12.0)

*Derived from established suicide risk assessments given to all clients.

+Caller identified concerns isolated with thematic analysis and natural language processing from narrative statements available in each call report.

Of the counselor-coded suicide risk indicators, which are assessed for all at-risk clients (e.g., our entire sample), difficulty sleeping (43%) and an expressed intent to die (57%) are the most common in this sample. Across help-seeking crisis concerns derived from NLP, interpersonal concerns (39.9%) and mental health (27%) were mentioned most frequently by users. Gender representation shifted with temperature extremes (men: 47.2% during heat; women: 61.2% during cold). Cold temperatures showed the highest proportions for most clinician-assessed risk indicators and help-seeking crisis concerns.

Temperature extremes exhibited the expected seasonal patterns: hot events (≥90th percentile) occurred predominantly from June to September, while cold events (≤10th percentile) clustered from December to February. 2023 had the highest occurrence of extreme heat events, while 2021–2022 experienced the most extreme cold, coinciding with Winter Storm Uri and other severe weather events (S1 Fig).

### 3.2 Distributed lag nonlinear models

**Fig 2:**
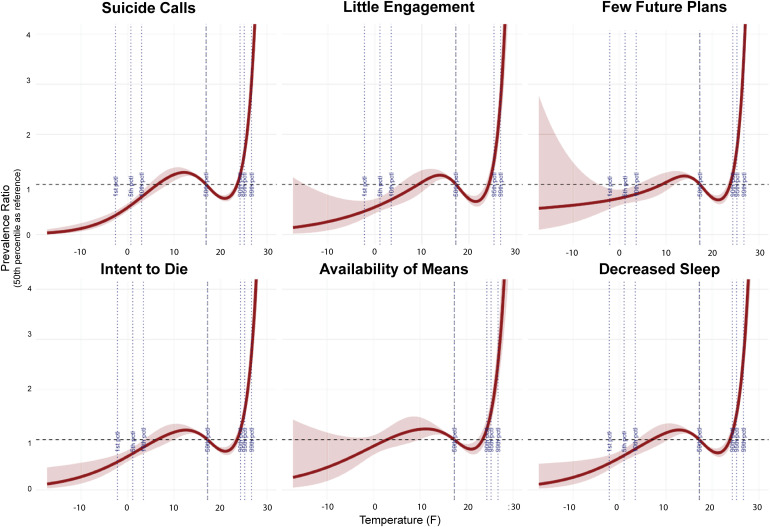
Prevalence ratio estimates for daily minimum temperature derived from distributed lag non-linear models using a 2-day lag. Prevalence estimates are in relation to median temperature (i.e., 50th percentile). Significant percentiles marked with dotted lines.

Suicide-related help-seeking showed a J-curve dose-response relationship with minimum temperature, with dramatic increases at extreme heat (Fig 2). Call volume increased 19% at the 90th percentile (PR = 1.19, 95% CI: 1.10-1.29), 55% at the 95th percentile (PR = 1.55, 95% CI: 1.44-1.68), and 166% at the 99th percentile (PR = 2.66, 95% CI: 2.42-2.92). These risk estimates translate to 7 fewer calls/100 callers at the coldest temperatures (Absolute Risk Difference (ARD: -7) and 19 additional calls per 100 calls at the hottest temperatures (ARD: 19) (Table 2).

**Table 2:** Prevalence ratio (PR) point estimates for suicide-related crisis conversations at minimum temperature extremes (e.g., 5th, 95th); results reported in reference to median temperature. Overall estimates (days 0-2) and lagged (days 0,1,2) estimates reported. Absolute risk difference (ARD) estimates represent expected increases and decreases in call volumes for each percentile per 100 calls, derived from prevalence ratio estimates. Stratified analysis for clinician-assessed suicide risk factors from the suicide risk assessment.

*Temperature* *Percentile*	*Cumulative* *(Lags 0–2)*		*Lag 0*		*Lag 1*		*Lag 2*	
	*PR* *(95th CI)*	*ARD* *(95th CI)*	*PR* *(95th CI)*	*ARD* *(95th CI)*	*PR* *(95th CI)*	*ARD* *(95th CI)*	*PR* *(95th CI)*	*ARD* *(95th CI)*
**Suicide Calls**								
1^st^	0.40 (0.34-0.48)	-7 (-7- -6)	0.54 (0.43-0.69)	-5 (-6 - -3)	1.21 (0.87-1.69)	2 (-1 - 8)	0.61 (0.48-0.77)	-4 (-6 - 3)
5^th^	0.55 (0.49-0.61)	-5 (-6 - -4)	0.66 (0.56-0.78)	−4 (-5 - -2)	1.14 (0.91-1.43)	2 (-1 - 5)	0.73 (0.62-0.86)	-3 (-4 - 2)
10^th^	0.67 (0.60-0.74)	-4 (-4 - -3)	0.75 (0.65-0.86)	-3 (-4- -2)	1.10 (0.91-1.32)	1 (-1 - 4)	0.81 (0.71-0.93)	−2 (-3 - 1)
90^th^	1.19 (1.10-1.29)	2 (1 - 3)	1.17 (1.01-1.37)	2 (0 - 4)	1.00 (0.81-1.22)	0 (-2 - 2)	1.02 (0.88-1.19)	0 (-1 - 2)
95^th^	1.55 (1.44-1.68)	6 (5 - 8)	1.33 (1.12-1.58)	4 (1 - 7)	1.02 (0.81-1.28)	0 (-2 - 3)	1.15 (0.96-1.36)	2 (0-4)
99^th^	2.66 (2.42-2.92)	19 (16 - 22)	1.71 (1.38-2.13)	8 (4 - 13)	1.07 (0.80-1.44)	1 (-2 - 5)	1.45 (1.16-1.80)	5 (2 -9 )
**Clinician Assessed Risk Factor: Availability of Means**								
1^st^	0.71 (0.56-0.90)	-1 (-2 - 0)	0.76 (0.54-1.07)	-3 (-5 - -1)	1.04 (0.65-1.66)	0 (-4 - 7)	0.90 (0.64-1.26)	-1 (-4 - 3)
5^th^	0.85 (0.72-0.996)	-1 (-1 - 0)	0.85 (0.67-1.07)	-2 (-4 - -1)	1.07 (0.77-1.50)	1 (-3 - 6)	0.94 (0.74-1.19)	-1 (-3 - 2)
10^th^	0.95 (0.81-1.11)	0 (-1 - 0)	0.90 (0.74-1.11)	-1 (-3 - 1)	1.09 (0.82-1.44)	1 (-2 - 5)	0.96 (0.78-1.19)	0 (-2 - 2)
90^th^	1.22 (1.08-1.38)	1 (0 - 2)	1.11 (0.87-1.42)	1 (-1 - 5)	1.05 (0.77-1.44)	1 (-3 - 5)	1.04 (0.82-1.32)	0 (-2 - 4)
95^th^	1.58 (1.39-1.79)	2 (2 - 3)	1.23 (0.93-1.63)	3 (-1 - 7)	1.06 (0.74-1.53)	1 (-3 - 6)	1.21 (0.91-1.59)	2 (-1 - 7)
99^th^	2.45 (2.09-2.87)	6 (4 - 7)	1.46 (1.02-2.09)	5 (0 - 12)	1.08 (0.68-1.72)	1 (-4 - 8)	1.55 (1.09-2.21)	6 (1 -14 )
**Clinician Assessed Risk Factor: Decreased Sleep**								
1^st^	0.52 (0.42-0.64)	-3 (-3 - -2)	0.62 (0.45-0.84)	-4 (-6 - 2)	1.49 (0.98-2.27)	5 (0 - 14)	0.56 (0.42-0.77)	-5 (-7 - 3)
5^th^	0.66 (0.57-0.76)	-2 (-3 - -1)	0.73 (0.59-0.90)	-3 (-5 - 1)	1.30 (0.97-1.74)	3 (0 - 8)	0.69 (0.56-0.86)	-3 (-5 - 2)
10^th^	0.76 (0.67-0.87)	-1 (-2 - -1)	0.81 (0.68-0.97)	-2 (-4 - 0)	1.19 (0.94-1.52)	2 (-1 - 6)	0.79 (0.66-0.94)	-2 (-4 - 1)
90^th^	1.17 (1.05-1.30)	1 (0 - 2)	1.08 (0.88-1.32)	1 (-1 - 4)	1.07 (0.83-1.40)	1 (-2 - 4)	1.01 (0.83-1.24)	0 (-2 - 3)
95^th^	1.49 (1.34-1.66)	3 (2 - 4)	1.22 (0.97-1.53)	2 (0 - 6)	1.09 (0.81-1.47)	1 (-2 - 5)	1.13 (0.90-1.42)	1 (-1 - 5)
99^th^	2.46 (2.16-2.80)	9 (7 - 11)	1.56 (1.16-2.10)	6 (2 - 12)	1.11 (0.75-1.65)	1 (-3 - 7)	1.42 (1.05-1.91)	5 (1 - 10)
**Clinician Assessed Risk Factor: Expressed Intent to Die**								
1^st^	0.51 (0.42-0.62)	-4 (-4 - -3)	0.66 (0.50-0.86)	-4 (-6 - 2)	1.01 (0.70-1.47)	0 (-3 - 5)	0.77 (0.59-1.01)	-3 (-5 - 0)
5^th^	0.65 (0.57-0.74)	-3 (-3 - -2)	0.75 (0.62-0.91)	-3 (-4 - 1)	1.01 (0.78-1.31)	0 (-3 - 4)	0.85 (0.71-1.03)	-2 (-3 - 0)
10^th^	0.76 (0.67-0.85)	-2 (-2 - -1)	0.82 (0.70-0.97)	-2 (-3 - 0)	1.01 (0.81-1.26)	0 (-2 - 3)	0.91 (0.77-1.07)	-1 (-3 - 1)
90^th^	1.21 (1.10-1.33)	2 (1 - 2)	1.14 (0.95 - 1.37)	1 (0 - 3)	1.04 (0.82-1.32)	0 (-1 - 2)	1.02 (0.84-1.22)	0 (-1 - 2)
95^th^	1.57 (1.42-1.72)	4 (3 - 5)	1.30 (1.06-1.59)	3 (1 - 7)	1.05 (0.80-1.37)	1 (-2 - 4)	1.15 (0.94-1.42)	2 (-1 - 5)
99^th^	2.63 (2.35-2.96)	12 (10 - 14)	1.67 (1.28-2.18)	8 (3 - 13)	1.05 (0.74-1.49)	1 (-3 - 5)	1.50 (1.15-1.95)	6 (2 - 11)
**Clinician Assessed Risk Factor: Little Engagement with Crisis Counselor**								
1st	0.57 (0.43-0.74)	-1 (-2 - -1)	0.57 (0.38-0.85)	-5 (-7 - 2)	1.52 (0.87-2.64)	6 (-1 - 18)	0.66 (0.44-0.99)	-4 (-6 - 0)
5th	0.64 (0.53-0.77)	-1 (-2 - -1)	0.69 (0.52-0.91)	-4 (-5 - 1)	1.10 (0.75-1.62)	1 (-3 - 7)	0.85 (0.64-1.12)	-2 (-4 - 1)
10th	0.70 (0.59-0.83)	-1 (-1 - 1)	0.77 (0.61-0.98)	−3 (-4 - 0)	0.94 (0.68-1.29)	-1 (-4 - 3)	0.97 (0.76-1.23)	0 (-3 - 3)
90th	1.12 (0.98-1.29)	0 (0 - 1)	1.09 (0.84-1.42)	1 (-2 - -5)	1.02 (0.72-1.44)	0 (-3 - 5)	1.01 (0.77-1.32)	0 (-3 - 4)
95th	1.43 (1.25-1.64)	1 (1 - 2)	1.20 (0.89-1.62)	2 (-1 - 7)	1.13 (0.76-1.66)	1 (-3 - 7)	1.06 (0.78-1.43)	1 (-2 - 5)
99th	2.35 (1.99-2.78)	5 (3 - 6)	1.47 (1.00-2.16)	5 (0 - 13)	1.38 (0.83-2.28)	4 (-2 - 14)	1.16 (0.78-1.72)	2 (-2 - 8)
**Clinician Assessed Risk Factor: Few to No Future Plans**								
1st	0.62 (0.49-0.78)	-2 (-2 - -1)	0.87 (0.62-1.21)	-2 (-4 - 2)	1.03 (0.65-1.63)	0 (-4 - 7)	0.70 (0.50-0.98)	-3 (-6 - 0)
5th	0.70 (0.60-0.83)	-1 (-2 - -1)	0.94 (0.74-1.19)	-1 (-3 - -2)	0.93 (0.67-1.29)	-1 (-4 - 3)	0.81 (0.64-1.02)	-2 (-4 - 0)
10th	0.77 (0.66-0.90)	-1 (-1 - 0)	0.99 (0.81-1.21)	0 (-2 - 2)	0.89 (0.68-1.17)	-1 (-4 - 2)	0.88 (0.71-1.08)	-1 (-3 - 1)
90th	1.26 (1.12-1.41)	1 (1 - 2)	1.15 (0.91-1.45)	2 (-1 - 5)	0.93 (0.69-1.25)	-1 (-4 - 3)	1.18 (0.94-1.49)	2 (-1 - 5)
95th	1.72 (1.53-1.93)	3 (2 - 4)	1.30 (1.00-1.69)	3 (0 - 8)	0.97 (0.69-1.37)	0 (-3 - 4)	1.36 (1.05-1.76)	4 (1 - 8)
99th	3.24 (2.82-3.72)	10 (8 - 12)	1.68 (1.20-2.34)	8 (2 - 15)	1.08 (0.70-1.66)	1 (-3 - 7)	1.79 (1.29-2.49)	9 (3 - 17)

All counselor-coded risk factors followed similar dose-dependent patterns: 12–26% increases at the 90th percentile (PR range: 1.12-1.26; ARD 0-2), 43–72% at the 95th percentile (PR range: 1.43-1.72; ARD: 1–6 additional calls per 100 calls), and 135–224% at the 99th percentile (PR range: 2.35-3.24, all p < 0.05; ARD: 5–19 additional calls per 100 calls). Few future plans (PR = 3.24, 95% CI: 2.82-3.72; ARD: 10 additional calls/100 calls) and expressed intent to die (PR = 2.63, 95% CI: 2.35-2.96; ARD: 12 additional calls/100 calls) showed the largest increases for extreme heat (Fig 2 and Table 2).

Effects were strongest on exposure day (lag 0), with a U-shaped pattern showing renewed risk at lag 2 (Table 2 and S2 Fig). This pattern persisted in the extended 10-day lag analyses (S3 Tables).

#### 3.2.1 Extreme cold associations.

Extreme cold showed opposing patterns to heat, with overall reductions in help-seeking: 33% decrease at the 10th percentile (PR = 0.67, 95% CI: 0.60-0.74), 45% at the 5th percentile (PR = 0.55, 95% CI: 0.49-0.61), and 60% at the 1st percentile (PR = 0.40, 95% CI: 0.34-0.48). These estimates translate to 7 fewer calls per 100 calls at the coldest temperatures (ARD: -7) and 4 fewer calls on days below the 10th percentile (ARD: -4).

**Fig 3:**
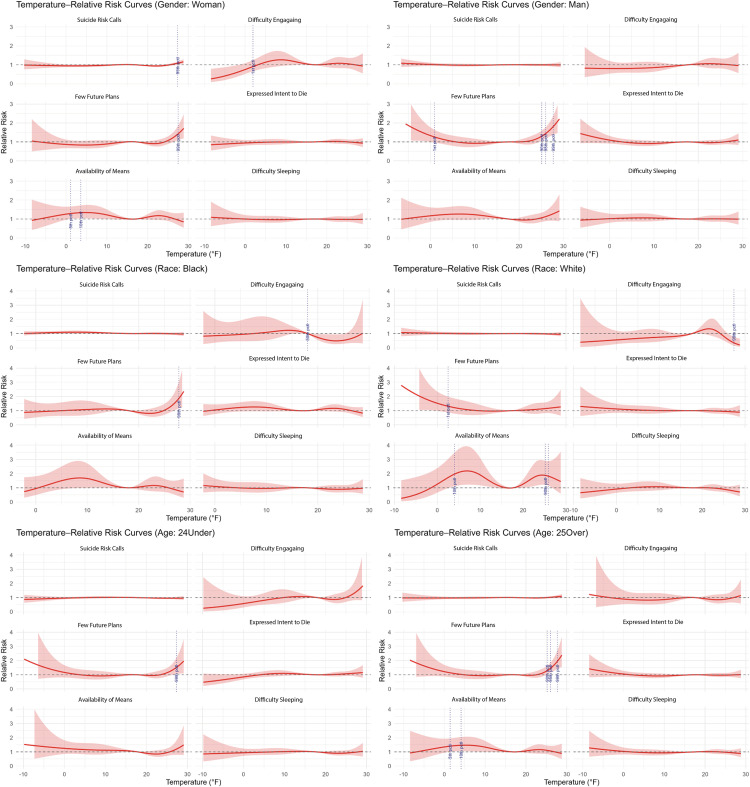
Prevalence ratio estimates for daily minimum temperature derived from distributed lag non-linear models using a 2-day lag across subgroups [age (≤24, ≧ 25), gender (men, women), and race (Black, white)]. Prevalence estimates are in relation to median temperature (i.e., 50th percentile).

However, subgroup analyses revealed important exceptions. During cold temperatures, availability of means increased 47% among adults (PR10th: 1.47, CI: 1.05-2.05) and 34% among women (PR10th: 1.34, CI: 1.04-1.73). Men showed 41% higher risk for few future plans during extreme cold (PR1st: 1.41, CI: 1.03-1.94). Effects were most pronounced on exposure day (Table 3, Fig 3 and S2 Table).

**Table 3:** Prevalence ratio point estimates for suicide-related crisis conversations at temperature extremes (e.g., 5th, 95th). Risk derived for subgroups [age (<25, > 25), gender [man, woman), and race (Black, white)].

Suicide Help-Seeking Calls																		
*Race Stratified*							*Age Stratified*						*Gender Stratified*					
Percentile	White			Black			Youth			Adults			Women			Men		
	PR	LCI	UCI	PR	LCI	UCI	PR	LCI	UCI	PR	LCI	UCI	PR	LCI	UCI	PR	LCI	UCI
1st	1.03	0.90	1.18	0.96	0.76	1.22	0.95	0.85	1.06	0.97	0.87	1.09	0.95	0.86	1.04	1.03	0.93	1.14
5th	1.01	0.92	1.11	1.15	0.95	1.39	0.98	0.91	1.06	0.98	0.90	1.06	0.94	0.88	1.01	1.00	0.93	1.08
10th	1.00	0.91	1.10	1.28	1.03	1.58	1.00	0.92	1.07	0.98	0.90	1.06	0.94	0.88	1.01	0.99	0.92	1.06
90th	0.98	0.91	1.05	1.12	0.96	1.31	0.96	0.90	1.02	1.00	0.94	1.07	0.98	0.93	1.03	1.00	0.94	1.06
95th	0.97	0.90	1.05	1.11	0.96	1.28	0.96	0.90	1.02	1.02	0.95	1.09	1.01	0.96	1.07	1.00	0.94	1.06
99th	0.96	0.86	1.07	1.07	0.91	1.27	0.96	0.88	1.05	1.06	0.96	1.17	1.08	1.00	1.17	0.99	0.91	1.08
**Means Available**																		
Percentile	White			Black			Youth			Adults			Women			Men		
	PR	LCI	UCI	PR	LCI	UCI	PR	LCI	UCI	PR	LCI	UCI	PR	LCI	UCI	PR	LCI	UCI
1st	0.80	0.33	1.97	0.90	0.45	1.79	1.28	0.81	2.04	1.29	0.85	1.95	1.19	0.85	1.67	1.15	0.80	1.65
5th	1.50	0.84	2.69	1.18	0.69	2.00	1.21	0.87	1.68	1.40	1.01	1.95	1.29	1.00	1.66	1.21	0.92	1.60
10th	1.97	1.11	3.48	1.53	0.92	2.57	1.17	0.84	1.63	1.47	1.05	2.05	1.34	1.04	1.73	1.25	0.95	1.65
90th	1.88	1.21	2.91	1.03	0.68	1.58	0.94	0.72	1.23	1.11	0.85	1.45	1.12	0.90	1.38	1.07	0.85	1.34
95th	1.81	1.14	2.88	0.93	0.58	1.50	1.01	0.76	1.35	1.07	0.80	1.43	1.06	0.84	1.33	1.13	0.89	1.43
99th	1.64	0.88	3.07	0.79	0.40	1.55	1.19	0.80	1.79	0.96	0.62	1.48	0.95	0.69	1.31	1.28	0.91	1.79
**Decreased Sleep**																		
Percentile	White			Black			Youth			Adults			Women			Men		
	PR	LCI	UCI	PR	LCI	UCI	PR	LCI	UCI	PR	LCI	UCI	PR	LCI	UCI	PR	LCI	UCI
1st	0.84	0.53	1.33	1.11	0.71	1.73	0.93	0.66	1.30	1.06	0.81	1.39	1.02	0.80	1.30	1.01	0.77	1.32
5th	0.95	0.70	1.30	1.05	0.74	1.48	0.96	0.76	1.21	1.01	0.81	1.25	0.99	0.83	1.18	1.03	0.85	1.26
10th	1.02	0.75	1.39	0.99	0.70	1.40	0.98	0.78	1.23	0.97	0.78	1.20	0.98	0.82	1.17	1.05	0.87	1.28
90th	0.90	0.71	1.14	0.90	0.69	1.19	0.92	0.76	1.10	0.97	0.82	1.15	0.98	0.85	1.13	1.01	0.86	1.18
95th	0.86	0.66	1.11	0.92	0.69	1.23	0.93	0.77	1.14	0.95	0.80	1.14	0.98	0.84	1.14	1.01	0.85	1.19
99th	0.78	0.54	1.13	0.95	0.63	1.42	0.98	0.74	1.29	0.92	0.71	1.18	0.98	0.79	1.21	1.00	0.79	1.27
**Expressed Intent to Die**																		
Percentile	White			Black			Youth			Adults			Women			Men		
	PR	LCI	UCI	PR	LCI	UCI	PR	LCI	UCI	PR	LCI	UCI	PR	LCI	UCI	PR	LCI	UCI
1st	1.17	0.81	1.68	1.02	0.72	1.45	0.79	0.59	1.05	1.09	0.87	1.36	0.92	0.76	1.12	1.16	0.93	1.45
5th	1.10	0.85	1.42	1.13	0.86	1.47	0.92	0.75	1.12	1.01	0.85	1.21	0.95	0.82	1.10	1.03	0.88	1.21
10th	1.06	0.82	1.38	1.22	0.94	1.59	1.00	0.83	1.21	0.96	0.80	1.15	0.97	0.84	1.12	0.96	0.82	1.13
90th	0.95	0.78	1.16	1.09	0.88	1.34	1.06	0.91	1.24	0.98	0.85	1.13	1.01	0.89	1.13	0.98	0.86	1.12
95th	0.94	0.76	1.16	1.02	0.81	1.28	1.08	0.92	1.27	0.98	0.85	1.14	0.99	0.87	1.12	1.00	0.87	1.15
99th	0.91	0.67	1.24	0.90	0.65	1.24	1.11	0.88	1.40	1.00	0.81	1.23	0.96	0.81	1.14	1.05	0.87	1.27
**Little Engagement with Crisis Counselor**																		
Percentile	White			Black			Youth			Adults			Women			Men		
	PR	LCI	UCI	PR	LCI	UCI	PR	LCI	UCI	PR	LCI	UCI	PR	LCI	UCI	PR	LCI	UCI
1st	0.52	0.20	1.36	0.84	0.34	2.11	0.55	0.27	1.12	0.93	0.51	1.69	0.60	0.37	0.98	0.80	0.51	1.27
5th	0.58	0.30	1.14	0.88	0.43	1.81	0.67	0.41	1.11	0.86	0.54	1.38	0.85	0.61	1.18	0.80	0.57	1.12
10th	0.64	0.34	1.20	0.95	0.46	1.97	0.78	0.48	1.26	0.83	0.52	1.32	1.04	0.76	1.42	0.81	0.58	1.11
90th	0.77	0.48	1.23	0.56	0.30	1.04	1.03	0.71	1.49	0.90	0.63	1.29	1.06	0.81	1.37	1.03	0.80	1.33
95th	0.59	0.35	1.01	0.65	0.33	1.29	1.13	0.77	1.66	0.94	0.65	1.37	1.03	0.78	1.36	1.02	0.78	1.33
99th	0.35	0.16	0.78	0.85	0.33	2.17	1.39	0.83	2.35	1.06	0.63	1.77	0.98	0.67	1.44	0.99	0.68	1.44
**Few Future Plans**																		
Percentile	White			Black			Youth			Adults			Women			Men		
	PR	LCI	UCI	PR	LCI	UCI	PR	LCI	UCI	PR	LCI	UCI	PR	LCI	UCI	PR	LCI	UCI
1st	1.80	1.05	3.10	0.90	0.50	1.64	1.25	0.81	1.93	1.31	0.90	1.91	0.90	0.65	1.24	1.41	1.03	1.94
5th	1.41	0.93	2.13	0.95	0.60	1.50	1.07	0.78	1.48	1.14	0.84	1.55	0.85	0.67	1.08	1.19	0.93	1.53
10th	1.22	0.80	1.87	1.00	0.63	1.59	0.99	0.71	1.37	1.02	0.75	1.40	0.83	0.66	1.06	1.05	0.82	1.36
90th	1.15	0.83	1.58	1.11	0.78	1.59	1.13	0.88	1.46	1.29	1.01	1.63	1.07	0.88	1.29	1.31	1.07	1.61
95th	1.17	0.84	1.64	1.36	0.94	1.97	1.25	0.96	1.63	1.45	1.14	1.85	1.17	0.96	1.43	1.44	1.17	1.77
99th	1.22	0.77	1.94	1.89	1.15	3.09	1.51	1.05	2.18	1.94	1.40	2.69	1.42	1.09	1.85	1.81	1.38	2.38

#### 3.2.2 Sensitivity analyses with average and maximum temperature.

Average temperature showed attenuated but similar patterns to minimum temperature, with suicide calls increasing 8% (PR = 1.08, 95% CI: 1.00-1.17), 18% (PR = 1.18, 95% CI: 1.09-1.28), and 49% (PR = 1.49, 95% CI: 1.32-1.67) at the 90th, 95th, and 99th percentiles, respectively (S4 Table).

Maximum temperature paradoxically showed protective effects at both extremes for suicide calls (PR: 0.50-0.89) and difficulty sleeping (PR: 0.60-0.88), supporting our focus on nighttime temperature as the primary exposure (S4 Table).

### 3.3 GAM help-seeking crisis concern theme analysis

**Fig 4:**
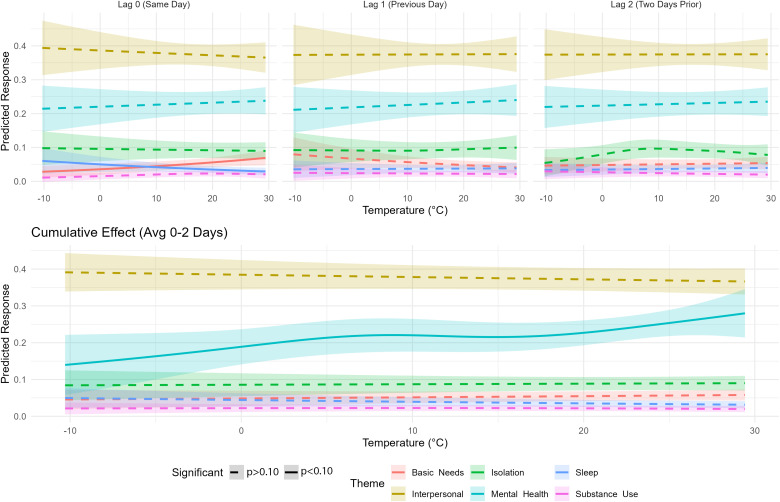
Temperature effects on crisis call themes derived from generalized additive models (GAMs) with penalized smoothing splines (k  = 8). Estimates derived by lag (days 0, 1, 2) and overall effect (0-2 day).

Help-seeking crisis concerns demonstrated distinct temperature relationships (Fig 4). Mental health calls exhibited the strongest association with a J-shaped non-linear pattern (EDF = 3.14, p = 0.09), increasing 20.9% at the 90th percentile compared to the 10th.

Basic needs showed a significant acute linear increase with temperature (EDF = 1.0, p = 0.02), rising 59.9% from the 10th to 90th percentiles on the day of exposure. Sleep-related help-seeking demonstrated a marginally significant linear pattern (EDF = 1.0, p = 0.08), with a 31.9% decrease at the 10th percentile on the day of exposure. Interpersonal concerns, isolation and substance use showed no significant temperature association (S5 Table).

#### 3.3.1 Sensitivity by demographic subgroups.

**Fig 5:**
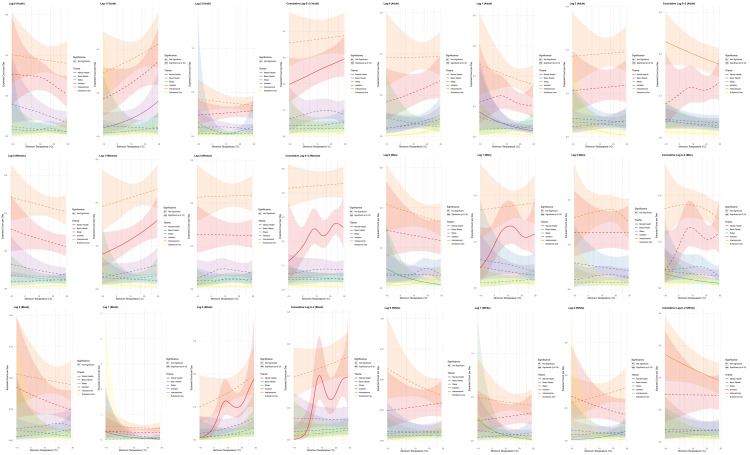
Subgroup temperature effects on crisis call themes derived from generalized additive models (GAMs) with penalized smoothing splines (k  = 8). Estimates derived as overall effect for lags 0-2.

Demographic groups showed distinct temperature vulnerabilities (Fig 5). During hot temperatures, mentions of mental health were higher among women (+25.9%, p:0.08), youth (+21.5%, p:0.09), and Black callers (+56.7%, p < 0.005) in the two days following a hot night. During cold temperatures, adults (-13.1% at 90th, p:0.09) and white callers (-17.5% at 90th, p:0.09) were more likely to mention interpersonal concerns in the two days following a cold night.

Lagged effects revealed group-specific patterns. Day-of effects were observed for men, who were more likely to mention substance use during hot temperatures (+137.4%, p:0.08) and sleep during cold temperatures (+40.1% at 10th, -59.9% at 90th, p:0.026). Adults were also associated with day-of associations, with mentions of substance use 325.7% higher at the 90th percentile (p:0.02). Youth and women were associated with delayed effects following a hot night, with youth (+109.1%, p:0.05) and women were more likely to mention mental health at the 90th percentile (+43.5%, p:0.04) the day after exposure to a hot night. Black callers were also associated with delayed effects, particularly two days after a hot night, with mentions related to mental health (+84.1%, p:0.01) and substance use (+801.7%, p:0.018) higher at the 90th percentile (S6 Table).

#### 3.3.2 Moderation by sleep.

The temperature × sleep interaction was significant at p < 0.10 (β = -0.0671, p = 0.060) and stratified analyses revealed important patterns, notably that sleep issues are most significantly associated with mental health between the 10th to 90th temperature percentiles (OR:1.11; p < 0.001). (Table 4).

**Table 4:** Sensitivity analysis of sleep by mental health moderation in response to cold temperatures (<10th percentile), normal temperatures (10-90th percentiles) and hot temperatures (>90th percentile). Estimates derived using generalized additive models with a 2-day lag structure and penalized smoothing splines (k = 8).

Percentile	# of conversations	Sleep Issues n (%)	Mental Health n (%)	Both Themes n (%)	Mental health & Sleep+	OR (95% CI)
<10th	1169	519 (44.4%)	312 (26.7%)	148 (12.7%)	28.5	1.18 (0.91-1.53)
10th-90th	9345	4001 (42.8%)	2520 (27.0%)	1128 (12.1%)	28.2	1.11* (1.02-1.22)
>90th	1170	499 (42.6%)	326 (27.9%)	139 (11.9%)	27.9	1.00 (0.77-1.29)

+Proportion of mental health conversations that mention sleep.

* p < 0.05.

## 4. Discussion

This study examines real-time suicide help-seeking during temperature extremes, combining time series analysis with natural language processing of crisis narratives to quantify risk magnitude and offer insights into precipitating contexts, a novel approach. Consistent with prior work [1,3], we found a dramatic dose-response relationship, with suicide-related help-seeking increasing 166% during extreme heat. Notably, basic needs crises, social isolation, mental health concerns, and all suicide risk indicators cluster during temperature extremes, providing mechanistic insights that are often unavailable within other health data sources (e.g., mortality). These findings support multiple theoretical pathways, including physiological (sleep disruption), behavioral (aggression/impulsivity), social (routine disruption), and structural (basic needs), while identifying actionable intervention targets for vulnerable populations.

### 4.1 Physiological pathway: Sleep disruption

The physiological pathway through sleep disruption emerges as particularly important. Extensive research indicates that sleep is one of the strongest longitudinal predictors of suicide risk [33], and evidence suggests that heat exposure disrupts sleep quality and duration [23,24]. We observed a marked increase in difficulty sleeping as a clinician-assessed suicide risk factor during extreme nighttime heat among help-seekers, adding additional evidence that poor and disrupted sleep during high temperatures is associated with increased suicidality. On the day of exposure, sleep was more likely to be mentioned as a context related to help-seeking during cold temperatures, and all callers were consistently more likely to mention sleep-related crisis concerns alongside mental health concerns across non-extreme temperature thresholds (e.g., between 10–90th), suggesting the sleep-suicide pathway is present among many suicide help-seekers and across temperature exposures. The sleep-suicide pathway is especially critical because sleep disruption impairs emotional regulation, decision-making, and stress resilience [51,52], all of which are protective factors against suicide [53,54].

In addition, the stronger associations with minimum versus average and maximum temperature provide crucial mechanistic insight, confirming nighttime heat as a distinct risk period. The increase in sleep difficulties during extreme minimum temperatures aligns with evidence linking sleep disruption to suicide risk [55] and demonstrates that public health responses focusing solely on daytime heat miss critical prevention opportunities. Louisiana’s accelerating nighttime warming trend, with record hot nights from 2015-2020 [30], demands immediate implementation of nighttime-specific interventions, including extended cooling center hours, utility disconnection moratoriums during heat events, and distribution of bedroom cooling units to vulnerable populations. These findings support emerging evidence that nighttime temperatures may be more relevant than daytime heat for various health outcomes [56].

### 4.2 Basic needs and motivational-volitional theory

During high temperatures clients exhibited significant basic needs stressors as a help-seeking context on the day of exposure and all clients were more likely to have access to lethal means (i.e., capability) up to two days after a high temperature event, offering exploratory evidence of the motivation-volitional theory during high temperatures. Paradoxically, cold weather, while associated with overall decreases in call volumes, was associated with increased basic needs stress among Black callers in exploratory population-level analyses; though results should be interpreted with care as race data had high missingness (i.e., ~ 79% missing). However, this finding could suggest that while cold temperatures are associated with lower call volumes, callers who are seeking help are reaching out for immediate crises (e.g., utilities, food assistance). Additional explanations include differential access to healthcare and essential services during winter weather events [57] and potential impacts to social well-being and interpersonal stressors [58].

Temperature-triggered staffing protocols represent a feasible extension of existing weather-responsive public health systems, such as heat warning systems and cold weather shelters [59]. Our results suggest the potential for 19 additional suicide-related calls per 100 calls following extremely hot nights, representing predictable increased demand. Many crisis hotlines already implement surge capacity protocols for predictable high-demand periods (e.g., hurricanes); integrating temperature forecasts into these protocols would leverage existing infrastructure. Utility disconnection moratoriums during extreme temperatures have been implemented in various jurisdictions (e.g., winter protections in northern states) and could be expanded to include extreme heat periods with relatively low implementation costs compared to their potential public health benefits [60]. The need for integrating social service and suicide-risk research is further evidenced by past work which highlights the efficacy of social service programs addressing basic needs (e.g., increased minimum wage, healthcare coverage) in reducing suicide ideation [61] and deaths [14,62]. The motivational-volitional theory stresses that burdensomeness and entrapment are precursors to active suicide risk [13], with stress related to essential needs potentially explaining higher rates of suicide [14,63]. Thus, affordable housing initiatives, higher minimum wages, and government subsidized programs (e.g., Supplemental Nutrition Assistance Program [SNAP]) also act as suicide prevention [14,62], offering numerous avenues for upstream intervention.

The prominence of basic needs themes during both hot and cold events indicates that infrastructure vulnerabilities (e.g., heating, power, shelter, food) may mediate temperature-suicide associations [64,65], emphasizing the importance of integrated suicide prevention approaches that leverage effective upstream interventions [66]. For example, programs that support utility assistance and moratoriums on utility disconnections during extreme hot and cold temperatures could also serve as suicide prevention, alleviating some of the economic burden associated with extreme temperatures [14,63,65]. Though not evaluated in this analysis, future work could employ quasi-experimental designs comparing jurisdictions that implement temperature-responsive policies to matched controls, measuring outcomes such as crisis call volume, emergency department visits for suicide-related concerns, and suicide attempts during extreme weather events. Prior work investigating extreme weather infrastructure failures suggests widespread difficulty accessing essential services (e.g., healthcare) [67], further illustrating the importance of free community resources, like 24/7 crisis and informational hotlines during periods of extreme temperatures. Our work adds preliminary evidence to the motivational-volitional theoretical model [13,14] in the context of hot and cold temperatures, emphasizing that the pathway between basic needs stressors and suicidality may be exacerbated during temperature extremes. Exploratory demographic differences exhibited at cold temperature extremes emphasize the importance of timely targeted interventions. Additional research is needed to understand how integrated upstream interventions can dually alleviate the economic and infrastructural burdens of extreme temperatures and reduce suicide risk [63]. Evidence is particularly needed for populations in unstable housing and resource-limited communities, as exposure to temperature extremes amplifies existing structural inequalities [68].

### 4.3 Temperature-aggression and behavioral pathways

Increased mentions of lethal means availability, expressed intent to die, and reporting few future plans during heat support the temperature-aggression hypothesis, which links high temperatures to increased impulsivity and aggression [9,10]. Men were associated with particularly elevated risk, reporting few future plans during both hot and cold temperature extremes, consistent with neurobiological effects of heat on impulsivity [16] and prior evidence of young men’s heightened vulnerability to heat-related interpersonal stressors and self-harm [20].

Substance use patterns provided additional evidence for behavioral pathways. Substance use can increase impulsivity and impair judgment, making it a key risk factor on the potential mechanistic pathway from extreme heat exposure to observed increases in suicidality [69]. Adults showed immediate increases in substance use mentions following high temperature exposure, while Black callers exhibited delayed effects, reporting mental health and substance use concerns in higher volumes two days post-exposure. White callers also exhibited increased mentions of substance use, though effects were delayed and observed only for cold temperatures. These temporal patterns may reflect maladaptive coping strategies (e.g., alcohol use) that initially mask but ultimately exacerbate mental health risks [70,71]. This pathway is particularly concerning given that 20% of suicide deaths involve opiates and 22% involve alcohol [72], and alcohol independently increases both impulsiveness [69,73] and heat-related mortality [74].

The routine activity theory posits that extreme temperature events change habitual behavior, thus increasing opportunities for interpersonal conflict [11]. From our analysis, social isolation in particular emerged as a novel but critical pathway, particularly for high temperatures. Notably, exploratory population-level results highlight that young people were associated with the starkest increases the day after high minimum temperature exposures. These exploratory findings extend prior research on heat-related isolation among older adults [75] to younger populations, suggesting that temperature extremes may disrupt social networks and support systems. At cold temperatures, exploratory subgroup results suggest that adults are associated with higher reports of isolation and both adults and white clients report more interpersonal concerns after cold nights, results in line with recent helpline-temperature analysis in Germany [58]. Our results provide additional context to the routine activity theory, whereby social isolation could be a contributing factor among young people particularly vulnerable to isolation and adults vulnerable to isolation and interpersonal stress, following high temperatures. At cold temperatures, our exploratory results suggest adults may also be vulnerable to social isolation and interpersonal stress associated with suicide-related help-seeking.

### 4.4 Exploratory population-specific insights

Our exploratory subgroup analyses reveal distinct risk profiles that require targeted interventions. In line with prior work [7,8], our analysis found women, Black clients, and youth exhibited distinctive risk profiles that may be more sensitive to high temperatures. Youth show heightened vulnerability to heat-related social isolations, highlighting the importance of peer relationships [76], and potential implications of limited coping resources [77]. Women were associated with stark increases in crisis concerns related to mental health in the days after exposure to a hot night, further extending understanding of social support importance, life-saving heat warning messaging and allocation of basic infrastructural resources (e.g., housing, power, A/C) in the backdrop of extreme weather exposure. Black callers exhibited cluster concerns, notably substance use and mental health, which were highest in the days following exposure to a hot night. Basic needs stress among Black clients at cold temperatures emphasizes the importance of integrated social service and suicide reduction efforts. Insight from male callers offers evidence of increased crisis severity during cold temperatures, particularly related to poor sleep, offering preliminary evidence of extreme cold as a suicide-related risk factor among this high suicide-risk population. These results are crucial in ensuring temperature-responsive crisis service messaging, outreach and availability during both hot and cold temperature events, though should be interpreted as exploratory given the high missingness present in demographic data.

### 4.5 Implications for integrated suicide prevention

Our results could be situated and applied to numerous suicide prevention frameworks, integrating predictable changes in suicide risk at temperature extremes into existing prevention approaches. For example, the *Gone Too Soon* and Center for Disease Control’s *Preventing Suicide: A Technical Package of Policy, Programs, and Practices* frameworks both call for interventions across socio-ecological levels that integrate support for essential services, like cash payments, which not only reduce entrapment related to suicide risk, but have wide-ranging benefits related to social wellness and cohesion [78–80]. These efforts could also be integrated with sleep promotion efforts, particularly given our findings of nighttime heat as a critical exposure period associated with increased risk. Targeting outreach efforts to reduce access to lethal means during extreme temperatures can also be integrated into numerous suicide prevention frameworks [79]. Leveraging digital interventions, like crisis hotlines and telehealth, has the potential to serve as a scalable intervention already in place to address numerous co-occurring crises [80].

### 4.6 Strengths and limitations

This analysis leveraged a rich, real-time crisis dataset providing unique insights into acute suicide risk during temperature extremes. The combination of rigorous time series modeling with natural language processing provides both quantitative risk estimates and qualitative context, which is rare in environmental health research. Exploratory subgroup analyses contribute unique insights for targeted interventions. To date, most literature has focused on suicide deaths and attempts [2]; leveraging help-seeking data contributes policy and practitioner-relevant results while expanding empirical understanding of suicide risk and extreme temperatures.

Several limitations should also be noted. Most significantly, demographic information was missing for 35–79% of calls, potentially introducing selection bias into subgroup analyses. Individuals who call crisis lines may differ systematically from those who do not seek help and our data capture only those callers meeting criteria for a suicide risk assessment (exhibiting suicide-related behaviors or reporting suicidal thoughts within the past 24 hours), representing acute crisis presentations rather than the full spectrum of suicidal ideation. Demographics of our sample show potential over-representation of youth, women, and Black help-seekers compared to the state of Louisiana as a whole. The study was conducted in Louisiana’s specific climate and demographic context, and results may not be applicable to other regions. Findings should be interpreted as reflecting temperature effects among crisis help-seekers in Louisiana rather than the general population with suicidal thoughts. However, the use of standardized suicide risk measures allows for the reproducibility of our analysis to other contexts. Our thematic analysis relied on keyword searching rather than manual coding, which may have resulted in the omission of nuanced content. Additionally, we cannot establish causal relationships between temperature exposure and suicide risk, only temporal associations. Future research should address these limitations through larger quasi-experimental designs to strengthen causal inference, validation of natural language processing approaches against clinical assessments, incorporation of richer covariate data on economic conditions and media exposure, and through multi-region studies that can enhance generalizability. Finally, while we used percentile-based temperature thresholds to account for local adaptation, we did not examine humidity-adjusted measures such as heat index or wet-bulb globe temperature. In Louisiana’s humid subtropical climate, these measures may provide additional insight into physiological thermal stress. Future research should investigate whether humidity-adjusted temperature metrics reveal different patterns in suicide-related help-seeking behaviors.

## 5. Conclusions

Extreme heat events are predictable mental health emergencies that must be prioritized in public health preparedness and climate adaptation planning. The sharp increase in crisis calls during high-temperature periods highlights the urgent need for weather-responsive crisis infrastructure, including dynamic staffing models and protocols addressing heat-specific risks like sleep disruption and social isolation. To prevent system overwhelm, coordination across crisis centers, emergency departments, and community clinics is essential. Mental health must be explicitly integrated into climate adaptation, with targeted interventions for youth, Black callers, women, and men based on differential vulnerability and need. Expanding heat messaging to address sleep and substance use, and investing in utility assistance and weatherization programs could help mitigate climate-related mental health inequities. As temperature extremes intensify, such efforts are not optional but critical to safeguarding population mental health.

## Supporting information

S1 Fig Time series of suicide-related crisis conversations and daily minimum temperature. Days that fall at or below the 1st, 5th, and 10th percentiles (extreme cold) are depicted in blues, while days at or above the 90th, 95th, and 99th percentiles (extreme heat) are depicted in reds.
(TIF)

S2 Fig Lagged (days 0,1,2) point estimates for each percentile across suicide-risk factors of interest. Prevalence ratio estimates for daily minimum temperature derived from distributed lag non-linear models using a 2-day lag. Prevalence estimates are in relation to median temperature (i.e., 50th percentile).
(TIF)

S1 Table Terms used to isolate themes in conversation notes. (DOCX)

S2 Table Subgroup lagged DLNM estimates (days 0, 1, 2); estimates are derived compared to median minimum temperature. (DOCX)

S3 Table Lagged (days 0–10) DLNM results; estimates are derived compared to median minimum temperature. (DOCX)

S4 Table Average and maximum temperature DLNM results, lag 0–2, estimates derived in relation to median average and maximum temperature, respectively. (DOCX)

S5 Table Lagged (0–2 days) generalized additive model (GAM) estimates. (DOCX)

S6 Table Subgroup overall (0–2 days) and lagged (days 0,1,2) generalized additive model (GAM) estimates for age (≤24, ≧ 25), gender (men, women) and race (Black, white). (DOCX)

## References

[1] Burke M, González F, Baylis P, Heft-Neal S, Baysan C, Basu S, et al. Higher temperatures increase suicide rates in the United States and Mexico. Nat Clim Chang. 2018;8(8):723–9. doi: 10.1038/s41558-018-0222-x

[2] Frangione B, Rodríguez Villamizar LA, Lang JJ, Colman I, Lavigne E, Peters C, et al. Short-term changes in meteorological conditions and suicide: a systematic review and meta-analysis. Environ Res. 2022;207:112230. doi: 10.1016/j.envres.2021.112230 34688638

[3] Gao J, Cheng Q, Duan J, Xu Z, Bai L, Zhang Y. Ambient temperature, sunlight duration, and suicide: a systematic review and meta-analysis. Sci Total Environ. 2019;646:1021–9. 30235587 10.1016/j.scitotenv.2018.07.098

[4] Kim Y, Kim H, Gasparrini A, Armstrong B, Honda Y, Chung Y, et al. Suicide and ambient temperature: a multi-country multi-city study. Environ Health Perspect. 2019;127(11):117007. doi: 10.1289/EHP4898 31769300 PMC6927501

[5] Zhou Y, Gao Y, Yin P, He C, Liu W, Kan H, et al. Assessing the burden of suicide death associated with nonoptimum temperature in a changing climate. JAMA Psychiatry. 2023;80(5):488–97. doi: 10.1001/jamapsychiatry.2023.0301 36988931 PMC10061320

[6] Helama S, Holopainen J, Partonen T. Temperature-associated suicide mortality: contrasting roles of climatic warming and the suicide prevention program in Finland. Environ Health Prev Med. 2013;18(5):349–55. doi: 10.1007/s12199-013-0329-7 23382022 PMC3773099

[7] Basu R, Gavin L, Pearson D, Ebisu K, Malig B. Examining the association between apparent temperature and mental health-related emergency room visits in California. Am J Epidemiol. 2018;187(4):726–35. doi: 10.1093/aje/kwx295 29020264

[8] Florido Ngu F, Kelman I, Chambers J, Ayeb-Karlsson S. Correlating heatwaves and relative humidity with suicide (fatal intentional self-harm). Sci Rep. 2021;11(1):22175. 34782650 10.1038/s41598-021-01448-3 PMC8593067

[9] Hipp JR, Curran PJ, Bollen KA, Bauer DJ. Crimes of opportunity or crimes of emotion? Testing two explanations of seasonal change in crime. Soc Forces. 2004;82(4):1333–72. doi: 10.1353/sof.2004.0074

[10] Mahendran R, Xu R, Li S, Guo Y. Interpersonal violence associated with hot weather. Lancet Planet Health. 2021;5(9):e571-2. 10.1016/S2542-5196(21)00210-2 34508676

[11] Cohn EG. Weather and crime. Br J Criminol. 1990;30(1):51–64.

[12] Watts N, Amann M, Arnell N, Ayeb-Karlsson S, Beagley J, Belesova K, et al. The 2020 report of The Lancet Countdown on health and climate change: responding to converging crises. Lancet. 2021;397(10269):129–70. doi: 10.1016/S0140-6736(20)32290-X 33278353 PMC7616803

[13] O’Connor RC, Kirtley OJ. The integrated motivational–volitional model of suicidal behaviour. Philos Trans R Soc Lond B Biol Sci. 2018;373(1754):20170268. doi: 10.1098/rstb.2017.0268 30012735 PMC6053985

[14] Sinyor M, Silverman M, Pirkis J, Hawton K. The effect of economic downturn, financial hardship, unemployment, and relevant government responses on suicide. Lancet Public Health. 2024;9(10):e802-6. 10.1016/S2468-2667(24)00152-X 39265607

[15] Kim SE, Kim Y, Hashizume M, Honda Y, Kazutaka O, Hijioka Y. Positive association of aggression with ambient temperature. Yale J Biol Med. 2023;96(2):189–96. 37396982 10.59249/RXZX5728 PMC10303254

[16] Meidenbauer KL, Schertz KE, Janey EA, Stier AJ, Samtani AL, Gehrke K, et al. Evidence for environmental influences on impulsivity and aggression. Urban For Urban Green. 2025;103:128594. doi: 10.1016/j.ufug.2024.128594

[17] Li J, Feng C, Yang J. Climate attribution of interpersonal violence: international evidence. Environ Res. 2023;236(Pt 2):116836. doi: 10.1016/j.envres.2023.116836 37543128

[18] Xu R, Xiong X, Abramson MJ, Li S, Guo Y. Ambient temperature and intentional homicide: a multi-city case-crossover study in the US. Environ Int. 2020;143:105992. 32738768 10.1016/j.envint.2020.105992

[19] Kubo R, Ueda K, Seposo X, Honda A, Takano H. Association between ambient temperature and intentional injuries: a case-crossover analysis using ambulance transport records in Japan. Sci Total Environ. 2021;774:145511. doi: 10.1016/j.scitotenv.2021.145511 33609821

[20] Zhao H, He L, Liu C, Shan X, Gui C, Zhang L, et al. Self-harm and interpersonal violence due to high temperature from the global burden of disease study 2019: a 30-year assessment. Environ Res. 2024;243:117826. doi: 10.1016/j.envres.2023.117826 38081341

[21] Le K. The impacts of extreme heat days on the prevalence of domestic abuse. Sage Open. 2025;15(1):21582440251317797. doi: 10.1177/21582440251317797

[22] Lai-Yi Wong E, Qiu H, Ho K-F, Wai-Ling Cheung A, Leung H, Chen FY, et al. Association of ambient temperature with social isolation among the community-dwelling Chinese older adults: a cross-sectional study in Hong Kong. Heliyon. 2025;11(1):e41721. doi: 10.1016/j.heliyon.2025.e41721 39866493 PMC11760327

[23] Chevance G, Minor K, Vielma C, Campi E, O’Callaghan-Gordo C, Basagaña X. A systematic review of ambient heat and sleep in a warming climate. Sleep Med Rev. 2024;75:101915. 38598988 10.1016/j.smrv.2024.101915

[24] Godwin JL, Lo YTE, Maude U, Timpson NJ, Northstone K. Extreme heat impacts on daily life and adaptive behaviours captured through lived experience. Environ Res Lett. 2025;20(5):054077. 10.1088/1748-9326/adcbc5 PMC7619029 42028462

[25] Obradovich N, Migliorini R, Paulus MP, Rahwan I. Empirical evidence of mental health risks posed by climate change. Proc Natl Acad Sci. 2018;115(43):10953–8. 30297424 10.1073/pnas.1801528115 PMC6205461

[26] Pigeon WR, Pinquart M, Conner K. Meta-analysis of sleep disturbance and suicidal thoughts and behaviors. J Clin Psychiatry. 2012;73(09):e1160-7. 10.4088/JCP.11r07586 23059158

[27] Sugg MM, Wertis L, Ryan SC, Green S, Singh D, Runkle JD. Cascading disasters and mental health: the February 2021 winter storm and power crisis in Texas, USA. Sci Total Environ. 2023;880:163231. 37023802 10.1016/j.scitotenv.2023.163231 PMC10874649

[28] Jiang N, Ban J, Guo Y, Zhang Y. The association of ambient temperature with depression in middle-aged and elderly people: a multicenter prospective repeat survey study in China. Environ Res Lett. 2022;17(8):084033.

[29] US Census. United States Census QuickFacts; 2021 [cited 2022 Jun 30]. U.S. Census Bureau QuickFacts: Louisiana. Available from: https://www.census.gov/quickfacts/LA

[30] Frankson R, Kunkel KE, Champsion SM, Nielsen-Gammon J. Louisiana state climate summary 2022 NOAA Technical Report NESDIS 150 [Internet]. NOAA NESDIS; 2022 [cited 2025 Jul 25]. Available from: https://statesummaries.ncics.org/chapter/la

[31] vialink.org. VIA LINK; 2025 [cited 2025 Jul 25]. VIA LINK | Listening • Understanding • Connecting. Available from: https://vialink.org/

[32] Müller K, Wickham H, Francois R, Bryan J, RStudio. tibble: Simple Data Frames [Internet]; 2023 [cited 2025 May 28]. Available from: https://cran.r-project.org/web/packages/tibble/index.html

[33] Liu RT, Steele SJ, Hamilton JL, Do QBP, Furbish K, Burke TA, et al. Sleep and suicide: a systematic review and meta-analysis of longitudinal studies. Clin Psychol Rev. 2020;81:101895. doi: 10.1016/j.cpr.2020.101895 32801085 PMC7731893

[34] Queiroz GD, Fay C, Hvitfeldt E, Keyes O, Misra K, Mastny T, et al. tidytext: Text mining using “dplyr”, “ggplot2”, and other tidy tools [Internet]; 2024 [cited 2025 May 28]. Available from: https://cran.r-project.org/web/packages/tidytext/index.html

[35] R Core Team. R: the R project for statistical computing [Internet]; 2022 [cited 2022 Jul 18]. Available from: https://www.r-project.org/

[36] Wickham H, Software P, PBC. stringr: Simple, Consistent Wrappers for Common String Operations [Internet]; 2023 [cited 2025 May 28]. Available from: https://cran.r-project.org/web/packages/stringr/index.html

[37] PRISM. PRISM Climate Group at Oregon State University [Internet]; 2023 [cited 2023 May 3]. Available from: https://prism.oregonstate.edu/explorer/

[38] Chambers J. Global and cross-country analysis of exposure of vulnerable populations to heatwaves from 1980 to 2018. Clim Change. 2020;163(1):539–58.

[39] Rennie J, Bell JE, Kunkel KE, Herring S, Cullen H, Abadi AM. Development of a submonthly temperature product to monitor near-real-time climate conditions and assess long-term heat events in the United States. J Appl Meteorol Climatol. 2019;58(12):2653–74. doi: 10.1175/jamc-d-19-0076.1

[40] US DHHS. COVID-19 Public Health Emergency [Internet]; 2023 [cited 2025 Jul 25]. Available from: https://www.hhs.gov/coronavirus/covid-19-public-health-emergency/index.html

[41] National Hurricane Center. HURDAT2: North Atlantic hurricane database (1851–present) [Data set]. [Internet]; 2024 [cited 2025 Oct 6]. Available from: https://www.nhc.noaa.gov/data/#hurdat

[42] Gasparrini A. A tutorial on the case time series design for small-area analysis. BMC Med Res Methodol. 2022;22(1):129. doi: 10.1186/s12874-022-01612-x 35501713 PMC9063281

[43] Min M, Shi T, Ye P, Wang Y, Yao Z, Tian S, et al. Effect of apparent temperature on daily emergency admissions for mental and behavioral disorders in Yancheng, China: a time-series study. Environ Health. 2019;18(1):98. 31771610 10.1186/s12940-019-0543-x PMC6880413

[44] Ulrich SE, Sugg MM, Guignet D, Runkle JD. Mental health disparities among maternal populations following heatwave exposure in North Carolina (2011–2019): a matched analysis. Lancet Reg Health Am [Internet]. 2025 [cited 2025 Apr 21];42. Available from: https://www.thelancet.com/journals/lanam/article/PIIS2667-193X(25)00008-0/fulltext 10.1016/j.lana.2025.100998 PMC11804822 39925466

[45] Yoo EH, Eum Y, Roberts JE, Gao Q, Chen K. Association between extreme temperatures and emergency room visits related to mental disorders: a multi-region time-series study in New York, USA. Sci Total Environ. 2021;792:148246. 34144243 10.1016/j.scitotenv.2021.148246

[46] Gasparrini A, Armstrong B, Scheipl F. Package ‘dlnm’ [Internet]; 2022. (Distributed Lag Non-Linear Models). Available from: https://cran.r-project.org/web/packages/dlnm/dlnm.pdf

[47] Gasparrini A. Modeling exposure–lag–response associations with distributed lag non-linear models. Stat Med. 2014;33(5):881–99. 24027094 10.1002/sim.5963 PMC4098103

[48] Liu C, Pan F, Li Y. A combined approach of generalized additive model and bootstrap with small sample sets for fault diagnosis in fermentation process of glutamate. Microb Cell Fact. 2016;15(1):132. doi: 10.1186/s12934-016-0528-1 27472926 PMC4966594

[49] Wood S. mgcv: Mixed GAM Computation Vehicle with Automatic Smoothness Estimation [Internet]; 2025 [cited 2025 Jul 25]. Available from: https://cran.r-project.org/web/packages/mgcv/index.html

[50] Vyssoki B, Kapusta ND, Praschak-Rieder N, Dorffner G, Willeit M. Direct effect of sunshine on suicide. JAMA Psychiatry. 2014;71(11):1231–7. doi: 10.1001/jamapsychiatry.2014.1198 25208208

[51] Palmer CA, Oosterhoff B, Bower JL, Kaplow JB, Alfano CA. Associations among adolescent sleep problems, emotion regulation, and affective disorders: findings from a nationally representative sample. J Psychiatr Res. 2018;96:1–8. doi: 10.1016/j.jpsychires.2017.09.015 28941378

[52] Wei X, Ma J, Liu S, Li S, Shi S, Guo X. The effects of sleep deprivation on risky decision making. Psychon Bull Rev. 2025;32(1):80–96. 39080188 10.3758/s13423-024-02549-6

[53] Colmenero-Navarrete L, García-Sancho E, Salguero JM. Relationship between emotion regulation and suicide ideation and attempt in adults and adolescents: a systematic review. Arch Suicide Res. 2022;26(4):1702–35. doi: 10.1080/13811118.2021.1999872 34821201

[54] Han J, Wong I, Christensen H, Batterham PJ. Resilience to suicidal behavior in young adults: a cross-sectional study. Sci Rep. 2022;12(1):11419. doi: 10.1038/s41598-022-15468-0 35794217 PMC9259642

[55] Littlewood DL, Kyle SD, Carter L-A, Peters S, Pratt D, Gooding P. Short sleep duration and poor sleep quality predict next-day suicidal ideation: an ecological momentary assessment study. Psychol Med. 2019;49(3):403–11. doi: 10.1017/S0033291718001009 29697037 PMC6331731

[56] He C, Kim H, Hashizume M, Lee W, Honda Y, Kim SE, et al. The effects of night-time warming on mortality burden under future climate change scenarios: a modelling study. Lancet Planet Health. 2022;6(8):e648-57. 10.1016/S2542-5196(22)00139-5 35932785

[57] Adepoju OE, Xu L, Chavez S, Dang P, Tipton M, Arguelles MP, et al. Back-to-Back Climate shocks and the mental health crisis: a Texas-sized surge in depression and anxiety ER visits. Am J Emerg Med. 2025;91:123–31. doi: 10.1016/j.ajem.2025.02.038 40049073

[58] Janzen B. Temperature and mental health: evidence from helpline calls. JAERE. 2025;12(6):1431–57.

[59] Chandra N SVS, Lee JKW. A systematic review of heat health warning systems: enhancing the framework towards effective health outcomes. Curr Environ Health Rep. 2025;12(1):31. doi: 10.1007/s40572-025-00496-5 40836170 PMC12367936

[60] Lusson K. Protecting access to essential utility service during extreme heat and climate change. National Consumer Law Center; 2024. (PROTECTING ACCESS TO ESSENTIAL UTILITY SERVICE).

[61] Austin AE, Frank M, Shanahan ME, Reyes HLM, Corbie G, Naumann RB. Association of state supplemental nutrition assistance program eligibility policies with adult mental health and suicidality. JAMA Network Open. 2023;6(4):e238415. doi: 10.1001/jamanetworkopen.2023.8415 PMC10105313 37058301

[62] Stack S. Contributing factors to suicide: political, social, cultural and economic. Prev Med. 2021;152(Pt 1):106498. doi: 10.1016/j.ypmed.2021.106498 34538366

[63] O’Connor RC, Kirtley OJ, Beurs D de. Preventing suicide: understanding the complex interplay between individual and societal factors. Lancet Public Health. 2024;9(10):e714-5. 10.1016/S2468-2667(24)00217-2 39265605

[64] Baek SU, Yoon JH. The mediating role of food insecurity in the relationship between income poverty and depressive symptoms and suicidal ideation: a nationwide study of Korean adults. Soc Sci Med. 2025;373:117972. 40188711 10.1016/j.socscimed.2025.117972

[65] Bentley R, Daniel L, Li Y, Baker E, Li A. The effect of energy poverty on mental health, cardiovascular disease and respiratory health: a longitudinal analysis. Lancet Reg Health West Pac. 2023;35:100734. doi: 10.1016/j.lanwpc.2023.100734 37424688 PMC10326697

[66] Hawton K, Pirkis J. Preventing suicide: a call to action. Lancet Public Health. 2024;9(10):e825-30. 10.1016/S2468-2667(24)00159-2 39265609

[67] Klinger C, Landeg O, Murray V. Power outages, extreme events and health: a systematic review of the literature from 2011-2012. PLoS Curr. 2014;6:ecurrents.dis.04eb1dc5e73dd1377e05a10e9edde673. doi: 10.1371/currents.dis.04eb1dc5e73dd1377e05a10e9edde673 PMC3879211 24459613

[68] Parsons ES, Jowell A, Veidis E, Barry M, Israni ST. Climate change and inequality. Pediatr Res. 2024:1–8. 10.1038/s41390-024-03443-6 PMC11772218 39075170

[69] Pirkis J, Bantjes J, Dandona R, Knipe D, Pitman A, Robinson J. Addressing key risk factors for suicide at a societal level. Lancet Public Health. 2024;9(10):e816-24. 10.1016/S2468-2667(24)00158-0 39265612

[70] Jhang H, Kim S, Kim K, Choi S, Choe S-A. Extreme ambient temperature and emergency healthcare service utilization due to substance use disorders: a systematic review and meta-analysis. Sci Rep. 2025;15(1):13582. doi: 10.1038/s41598-025-98247-x 40253512 PMC12009368

[71] Poorolajal J, Haghtalab T, Farhadi M, Darvishi N. Substance use disorder and risk of suicidal ideation, suicide attempt and suicide death: a meta-analysis. J Public Health. 2016;38(3):e282-91. 10.1093/pubmed/fdv148 26503486

[72] SAMHSA. Substance use and suicide: a nexus requiring a public health approach. In Brief [Internet]. Substance Abuse and Mental Health Services Administration; 2015. Available from: https://library.samhsa.gov/sites/default/files/sma16-4935.pdf

[73] Ryan SA, Kokotailo P, Committee on Substance Use and Prevention, Camenga DR, Patrick SW, Plumb J, et al. Alcohol use by youth. Pediatrics. 2019;144(1):e20191357. doi: 10.1542/peds.2019-1357 31235608

[74] Thompson R, Hornigold R, Page L, Waite T. Associations between high ambient temperatures and heat waves with mental health outcomes: a systematic review. Public Health. 2018;161:171–91. doi: 10.1016/j.puhe.2018.06.008 30007545

[75] Fang B, Zhang Q. Heatwaves and its impact on the depressive symptoms among Chinese community-dwelling older adults: examining the role of social participation. Arch Gerontol Geriatr. 2025;129:105668. doi: 10.1016/j.archger.2024.105668 39488030

[76] Cui S, Cheng Y, Xu Z, Chen D, Wang Y. Peer relationships and suicide ideation and attempts among Chinese adolescents. Child Care Health Dev. 2011;37(5):692–702. doi: 10.1111/j.1365-2214.2010.01181.x 21198776

[77] Chapman-Hilliard C, Pelham T, Mollo V, Henry P, Miller B, Yankura J, et al. Clinical utility of depression measures and symptoms: implications for suicide risk assessment in high risk, resource limited youth populations. Suicide Life Threat Behav. 2025;55(1):e13068. doi: 10.1111/sltb.13068 38411306 PMC13542460

[78] Stone DM, Holland KM, Bartholow BN, Crosby AE, Davis SP, Wilkins N. Preventing suicide: a technical package of policies, programs, and practice [Internet]; 2017 [cited 2025 Nov 2]. Available from: https://stacks.cdc.gov

[79] DeBeer B, Mignogna J, Talbot M, Villarreal E, Mohatt N, Borah E, et al. Suicide prevention programming: comparing four prominent frameworks. Psychiatr Serv. 2024;75(8):789–800. doi: 10.1176/appi.ps.20230173 38807579

[80] O’Connor RC, Worthman CM, Abanga M, Athanassopoulou N, Boyce N, Chan LF, et al. Gone Too Soon: priorities for action to prevent premature mortality associated with mental illness and mental distress. Lancet Psychiatry. 2023;10(6):452–64. doi: 10.1016/S2215-0366(23)00058-5 37182526

