# Supplementary figures and images for: Temperature extremes contribute to suicide-related help-seeking through multiple pathways: Evidence from crisis hotline data (2019–2023)
Source: PLOS Ment Health. 2026 Feb 11;3(2):e0000501. doi: 10.1371/journal.pmen.0000501 (PMC12893560; doi:10.1371/journal.pmen.0000501)

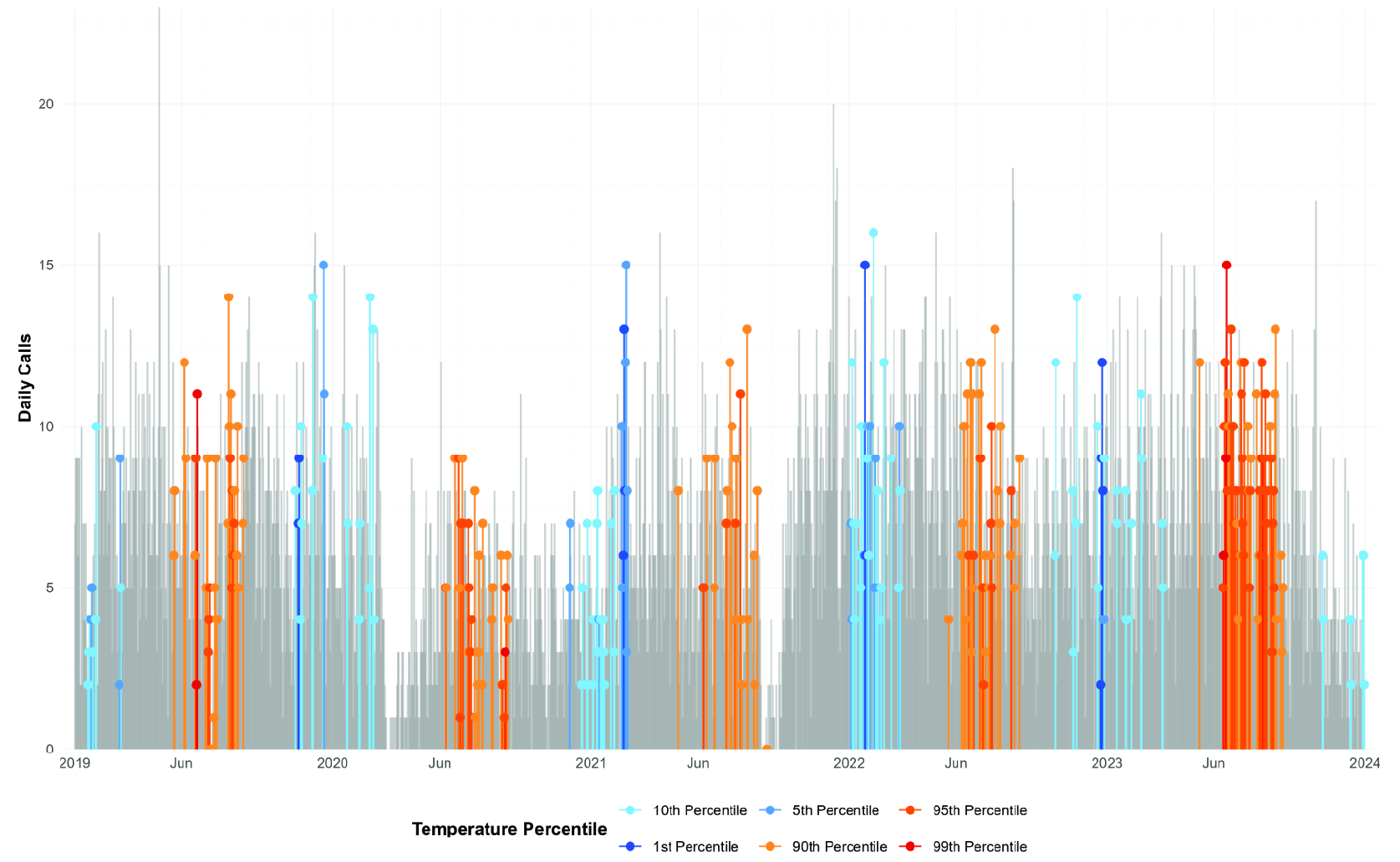

Supplement: S1 Fig — Days that fall at or below the 1st, 5th, and 10th percentiles (extreme cold) are depicted in blues, while days at or above the 90th, 95th, and 99th percentiles (extreme heat) are depicted in reds. (TIF) [file pmen.0000501.s001.tif]

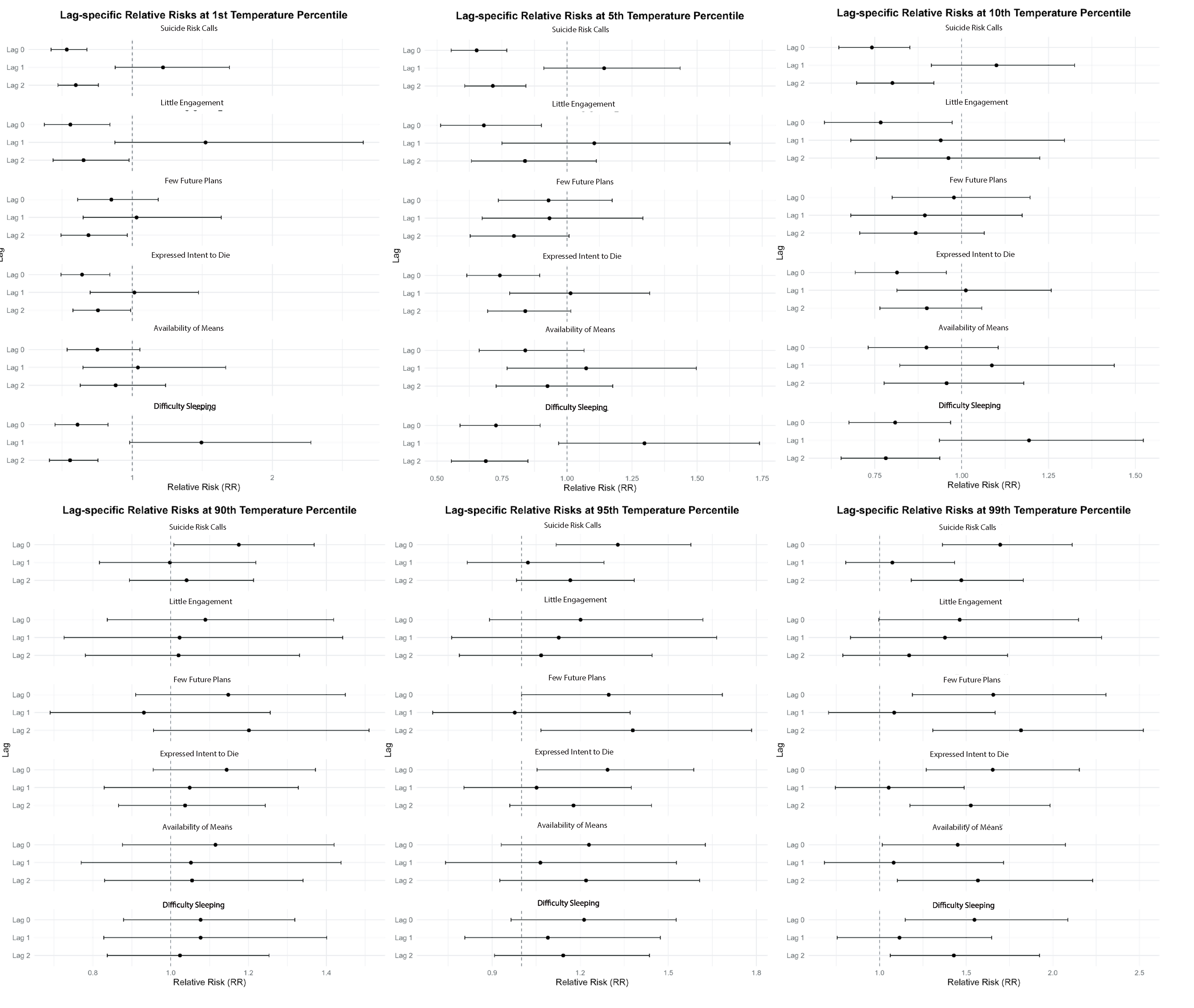

Supplement: S2 Fig — Prevalence ratio estimates for daily minimum temperature derived from distributed lag non-linear models using a 2-day lag. Prevalence estimates are in relation to median temperature (i.e., 50th percentile). (TIF) [file pmen.0000501.s002.tif]
