# Supplementary material for: Temperature extremes contribute to suicide-related help-seeking through multiple pathways: Evidence from crisis hotline data (2019–2023)
Source: PLOS Ment Health. 2026 Feb 11;3(2):e0000501. doi: 10.1371/journal.pmen.0000501 (PMC12893560; doi:10.1371/journal.pmen.0000501)
Supplement: S1 Table — (DOCX) [file pmen.0000501.s003.docx]

S1 Table. Terms used to isolate themes in conversation notes.

| Basic = "House", "Home", "Unhoused", "Homeless", "Homelessness",  "Water recently disconnected", "Power recently disconnected", "Gas recently disconnected",  "Housing", "Shelter", "Evict", "Evicted", "Place to live",  'food', 'hungry', 'hunger', 'meal', 'meals', 'eat', 'food pantry', 'food bank', 'soup kitchen',  'groceries', 'snap', 'ebt', 'food stamps',"Job", "Work", "Finances", "Lost work", "Lost job",  "Paycheck", "Boss", "Financial hardship", "Financial", "unemployed",  'water', 'thirsty', 'dehydrated', 'drinking water', 'hydration',  'clothes', 'clothing', 'coat', 'jacket', 'shoes', 'boots', 'socks', 'blanket',  'sleeping bag', 'winter clothes', 'dress', 'dressed', 'outfit',  'shower', 'bath', 'clean', 'hygiene', 'soap', 'shampoo', 'toothbrush',  'toothpaste', 'deodorant', 'sanitation', 'homeless', 'homelessness', 'unhoused', 'unsheltered',  'street', 'sleeping outside', 'sleeping in car', 'living in car', 'tent', 'encampment', 'camp', 'camping',  'life stressors',"heat", "hot", "heatwave",  "heat wave", "overheat", "overheating", "swelter", "sweltering", "burning up", "boiling",  "scorching", "searing", "baking", "sun", "sunburn", "sunstroke", "heatstroke", "heat stroke",  "heat exhaustion", "dehydration", "dehydrated", "thirsty", "parched", "sweating", "sweat",  "sweaty", "ac", "a/c", "air conditioning", "air conditioner", "cooling", "fan", "cool",  "cooling center", "cooling shelter", "cold", "freezing", "frigid", "freeze", "frost", "frozen",  "icy", "ice", "snow", "blizzard", "chilly", "chill", "hypothermia", "frostbite", "shivering",  "blanket", "heater", "heating", "Warm", "warmth", "coat", "jacket", "winter", "temperature drop",  "below freezing", "cold snap", "winter storm", "winter advisory", "winter warning", "winter watch",  "no heat", "broken heater", "heater broken", "heating broken", "no heating", "freezing temperatures",  "cold temperatures", "too cold", "extremely cold", "unbearably cold", "wind chill", 'a c', 'a / c', 'power',  'no power', 'no heat', 'no electricity', 'outage' |
| --- |
| Mental Health = "Mental Health", "Mental Illness", 'toks', 'si',"Anxiety",  "Anxious", "Stress", "Worried", "Nervous", "Mood", "Bipolar",  "Schizophrenia", "ADHD", "OCD", "Depression", "Mood disorder", "Psychosis",  "BPD", "Personality disorder", "Depressed", "depressive", "Hopeless/Helpless",  "Hopeless", "Hopelessness", "Helpless", "Helplessness", "No selfworth",  "Selfworth", "Nothing will ever get better", "Overwhelm", "Overwhelmedness",  "Overwhelmed", "Overwhelming", 'anxiety', 'depression', 'stress', 'trauma','mental',  'psychiatric', 'emotional', 'distress',  ' medication, disorder', 'schizophrenia', 'bipolar', 'psychosis', 'insomnia', 'panic',  'afraid', 'fear', 'nervous', 'worry', 'paranoid',  'hallucinating', 'voices', 'delusion', 'ptsd', 'ocd', 'adhd', 'dementia', 'alzheimer', 'anxious',  'mental illness', 'mental health', 'mental breakdown', 'psychological', 'ptsd', 'ocd',  'adhd', 'dementia', 'alzheimer', 'anxious', 'depressed',  'manic', 'mania', 'mood disorder', 'behavioral health', 'cognitive',  'mood swings', 'irritable', 'agitated', 'restless',  'nervous', 'panic', 'afraid', 'fear', 'worry', 'paranoid',  'hallucinating', 'voices', 'delusion', 'insomnia', 'nightmares', 'flashback', 'disoriented',  'confused', 'irritable', 'agitated', 'restless',  'overwhelmed', 'therapist', 'counselor', 'psychiatrist', 'psychologist', 'prescription',  'treatment', 'support group',' therapy session',  'counseling session', 'group therapy', 'inpatient', 'outpatient', 'psychiatric hospital', 'crisis center',  'self harm', 'selfharm', 'self-harm', 'hearing voices', 'hallucinating' |
| Substance = "Substance", "Addict", "Addiction", "Addictive", "Drugs", "Alcohol", "Opioids", "Heroin",  "DUI", "Alcoholism", "Recovery", "Booze", "Overdose", "Overdosing",  'substance', 'substance abuse', 'substance use', 'addiction', 'addicted', 'alcohol', 'alcoholic', 'alcoholism', 'drug', 'drugs', 'overdose', 'withdrawal', 'detox', 'rehab', 'sober', 'using', 'heroin', 'cocaine', 'relapse'), |
| Isolation = "Isolation", "Lonely", "Loneliness", "No support", "No support system",  "Feels like nobody cares", "No friends", "No family", "Feels distance", "Alone",  "isolated", "alone", "lonely", "loneliness", "isolation", "secluded",  "detached", "disconnected", "social withdrawal", "alienated", "socially isolated",  "excluded", "withdrawn", "social isolation", "shunned", "forlorn",  "abandoned", "unwanted", "neglected", "isolating", "disengaged", "ostracized",  "solitude", "outcast", "separation", "cut off",  "unsociable", "seclusion", "lack of connection", 'no connection', "no support", "no friends",  "no social support", "no contact", "social detachment", "antisocial", "detached from others", "cut off from society", 'no one loves' |
| Interpersonal = "Interpersonal", "Relationship", "Relationships", "Broke up", "Break-up", "Break up",  "Broke-Up", "Broken-up", "Broken up", "Girlfriend", "Boyfriend", "Cheated on",  "Cheating on", "GF", "BF", "Family", "Partner", "Fiancee", "ex-fiancee", "Spouse",  "Husband", "Wife", "Divorce", "Lost custody", "custody", "Sister", "Brother", "Sibling",  "Niece", "Nephew", "Parent", "Parents", "Mom", "Dad", "Mother", "Father", "Fiance",  "Newborn", "Pregnant", "Surrogate", "Marriage", "Wedding", "Kid", "Kids", "Child",  "Children", "Grandkids", "Grandchild", "Grandchildren", "Stepdad", "Stepmom", "Abortion",  "Grandma", "Grandpa", "Step father", "stepfather", "Social", 'Friend', "Friends", "Boys", "Girls",  'fight', 'roommate', 'poor relationships', "Trauma", "Abuse", "Past trauma", "Death", "Abusive", "Assault", "Rape", "Witnessed",  "Drowning", "Abused", "Murdered", "Killed", "Childhood abuse", "Abused as a child",  "Abusive parent", "Abusive dad", "Abusive stepdad", "Abusive mom", "Abusive stepmom", "Incest",  "Traumatic", "Since the age of", 'since he was', 'since she was', "mistreatment", "PTSD", "PTS", "Posttraumatic stress", "Grief",  "Grieve", "Grieving", "Pass away", "Died", "DCFS", "childhood abuse", "physical abuse",  "emotional abuse", "verbal abuse", "neglect", "sexual abuse", "domestic abuse",  "childhood trauma", "maltreatment", "emotional neglect", "physical neglect", "neglected childhood",  "abandoned", "deprivation", "lack of care", "unattended", "family violence", "parental separation",  "divorce", "family conflict", "domestic violence", "parental substance abuse",  "parental mental illness", "inconsistent caregiving", "childhood bullying", "peer bullying",  "school bullying", "verbal bullying", "physical bullying", "social exclusion", "emotional bullying",  "Cyberbullying", "parental divorce", "separation from parents", "parental abandonment",  "loss of parent", "parental estrangement", "drug use in childhood", "Alcoholism", "drug exposure",  "parental addiction", "substance abuse in the home", "psychological problems in parents",  "mental health struggles", "parental depression", "parental anxiety", "mental illness in childhood",  "physical illness in childhood", "chronic illness", "disability", "health struggles",  "hospitalization", "medical trauma", "parental death", "loss of a parent",  "death of a family member", "bereavement", "mourning", "traumatic loss", "unstable household",  "unpredictable environment", "inconsistent living conditions", "moving homes frequently",  "witnessing violence", "seeing violence", "exposure to violence", "witnessing abuse",  "witnessing trauma", "seeing physical violence", "emotional trauma",  "post-traumatic stress disorder (PTSD)", "dissociation", "attachment disorder",  "insecure attachment","attachment trauma", "parent-child disconnect", "abandonment issues", "inconsistent parenting","neglectful parenting", "emotionally unavailable parent", "harsh discipline", "parental criticism", "overprotective parenting", "victimized childhood", "trauma survivor", "childhood victim",  "victimized by family", "victim of neglect", "victim of abuse", "abandoned child",  "behavioral problems", "conduct disorders", "impulse control issues", "aggressive behavior", 'trigger', 'triggered' |
| Sleep = "sleep", "nightmare", "can't sleep", "cant sleep", "trouble sleeping", "sleeping", "insomnia",  "sleep deprivation", "restless", "sleep disorder", "dreams", "sleep apnea", "sleeplessness",  "night terrors", "fatigue", "tired", "exhausted", "drowsy", "sleepiness", "lack of sleep",  "sleep problems", "insomniac", "midnight awakening", "circadian rhythm", "sleep cycle",  "early waking", "sleep disturbances", "oversleeping", "sleeping pills", "narcolepsy",  "daytime sleepiness", "sleeping pattern", "restless legs", "sleepwalking", "sleep paralysis",  "unrefreshing sleep", "snoring", "loud breathing", "sleep hygiene", "sleep consultant",  "bedtime routine", "deep sleep", "REM sleep", "sleep study", "polysomnography", "melatonin",  "sleep medication", "CPAP", "sleep clinic", "chronic fatigue", "tiredness", "dozing off",  "falling asleep", "waking up in the middle of the night", "hypersomnia", "sleep talking",  "sleep phases", "sleep consistency", "poor sleep quality", "sleep monitoring", "sleep tracker",  "wakefulness", "sleep interruption", "sleep debt", "shift work sleep disorder", "jet lag",  "microsleep", "sleep deprivation symptoms", "chronic insomnia", "sleep fragmentation",  "sleep latency", "sleep rhythm", "sleep disorders" |
