# Supplementary material for: Temperature extremes contribute to suicide-related help-seeking through multiple pathways: Evidence from crisis hotline data (2019–2023)
Source: PLOS Ment Health. 2026 Feb 11;3(2):e0000501. doi: 10.1371/journal.pmen.0000501 (PMC12893560; doi:10.1371/journal.pmen.0000501)
Supplement: S2 Table — (DOCX) [file pmen.0000501.s004.docx]

S2 Table. Subgroup lagged DLNM estimates (days 0, 1, 2); estimates are derived compared to median minimum temperature.

| Percentile | Lag | RR | LCI | UCI | RR | LCI | UCI | RR | LCI | UCI | RR | LCI | UCI | RR | LCI | UCI | RR | LCI | UCI |
| --- | --- | --- | --- | --- | --- | --- | --- | --- | --- | --- | --- | --- | --- | --- | --- | --- | --- | --- | --- |
|  |  | 25 Over | | | 24 Under | | | White | | | Black | | | Men | | | Women | | |
| Suicide Calls | | | | | | | | | | | | | | | | | | | |
| 1% | 0 | 1.02 | 0.93 | 1.12 | 0.94 | 0.86 | 1.02 | 1.13 | 1.01 | 1.25 | 0.98 | 0.88 | 1.10 | 1.01 | 0.92 | 1.11 | 0.97 | 0.89 | 1.05 |
|  | 1 | 0.97 | 0.87 | 1.07 | 1.01 | 0.92 | 1.11 | 0.94 | 0.84 | 1.06 | 1.07 | 0.95 | 1.19 | 1.02 | 0.93 | 1.13 | 1.02 | 0.93 | 1.12 |
|  | 2 | 0.99 | 0.90 | 1.08 | 1.00 | 0.92 | 1.09 | 0.98 | 0.88 | 1.09 | 0.98 | 0.89 | 1.08 | 1.00 | 0.91 | 1.09 | 0.96 | 0.89 | 1.04 |
| 5% | 0 | 1.01 | 0.94 | 1.08 | 0.97 | 0.91 | 1.03 | 1.06 | 0.99 | 1.14 | 1.00 | 0.92 | 1.08 | 1.02 | 0.95 | 1.08 | 0.97 | 0.91 | 1.03 |
|  | 1 | 0.98 | 0.91 | 1.06 | 1.02 | 0.95 | 1.09 | 0.97 | 0.90 | 1.05 | 1.06 | 0.98 | 1.15 | 1.01 | 0.94 | 1.08 | 1.02 | 0.95 | 1.09 |
|  | 2 | 0.99 | 0.92 | 1.06 | 0.99 | 0.93 | 1.06 | 0.99 | 0.92 | 1.06 | 1.00 | 0.93 | 1.08 | 0.98 | 0.92 | 1.05 | 0.96 | 0.91 | 1.02 |
| 10% | 0 | 1.00 | 0.94 | 1.06 | 0.99 | 0.93 | 1.05 | 1.03 | 0.96 | 1.10 | 1.01 | 0.94 | 1.08 | 1.02 | 0.96 | 1.08 | 0.97 | 0.91 | 1.02 |
|  | 1 | 0.99 | 0.92 | 1.06 | 1.02 | 0.96 | 1.09 | 0.98 | 0.92 | 1.05 | 1.05 | 0.98 | 1.13 | 0.99 | 0.93 | 1.06 | 1.02 | 0.96 | 1.08 |
|  | 2 | 0.99 | 0.93 | 1.06 | 0.99 | 0.93 | 1.05 | 0.99 | 0.93 | 1.06 | 1.02 | 0.95 | 1.09 | 0.98 | 0.92 | 1.04 | 0.96 | 0.91 | 1.02 |
| 90% | 0 | 0.99 | 0.92 | 1.07 | 0.99 | 0.93 | 1.05 | 1.02 | 0.95 | 1.10 | 0.96 | 0.89 | 1.05 | 1.08 | 1.01 | 1.16 | 0.95 | 0.89 | 1.02 |
|  | 1 | 1.04 | 0.95 | 1.13 | 1.00 | 0.93 | 1.08 | 0.98 | 0.90 | 1.07 | 1.08 | 0.98 | 1.18 | 0.98 | 0.91 | 1.07 | 1.02 | 0.95 | 1.10 |
|  | 2 | 0.97 | 0.90 | 1.05 | 0.97 | 0.91 | 1.03 | 0.97 | 0.90 | 1.05 | 0.96 | 0.88 | 1.04 | 0.93 | 0.87 | 1.00 | 1.00 | 0.94 | 1.07 |
| 95% | 0 | 1.00 | 0.91 | 1.09 | 0.99 | 0.92 | 1.07 | 1.03 | 0.95 | 1.12 | 0.97 | 0.88 | 1.07 | 1.10 | 1.01 | 1.19 | 0.96 | 0.89 | 1.03 |
|  | 1 | 1.04 | 0.94 | 1.15 | 1.01 | 0.93 | 1.11 | 0.97 | 0.88 | 1.07 | 1.08 | 0.97 | 1.21 | 0.97 | 0.89 | 1.07 | 1.04 | 0.95 | 1.14 |
|  | 2 | 0.99 | 0.91 | 1.08 | 0.95 | 0.88 | 1.03 | 0.97 | 0.89 | 1.06 | 0.95 | 0.86 | 1.04 | 0.93 | 0.86 | 1.01 | 1.02 | 0.94 | 1.10 |
| 99% | 0 | 1.00 | 0.89 | 1.14 | 1.00 | 0.90 | 1.12 | 1.04 | 0.93 | 1.17 | 0.98 | 0.86 | 1.11 | 1.12 | 1.00 | 1.25 | 0.97 | 0.87 | 1.08 |
|  | 1 | 1.03 | 0.89 | 1.19 | 1.03 | 0.91 | 1.16 | 0.94 | 0.82 | 1.08 | 1.08 | 0.93 | 1.25 | 0.95 | 0.83 | 1.09 | 1.08 | 0.95 | 1.22 |
|  | 2 | 1.02 | 0.90 | 1.16 | 0.93 | 0.83 | 1.03 | 0.98 | 0.87 | 1.10 | 0.93 | 0.81 | 1.05 | 0.93 | 0.83 | 1.05 | 1.04 | 0.94 | 1.16 |
| Means Available | | | | | | | | | | | | | | | | | | | |
| 1% | 0 | 1.29 | 0.90 | 1.84 | 1.22 | 0.84 | 1.78 | 1.08 | 0.52 | 2.26 | 1.49 | 0.81 | 2.73 | 1.15 | 0.82 | 1.60 | 1.16 | 0.86 | 1.56 |
|  | 1 | 0.95 | 0.64 | 1.42 | 1.09 | 0.72 | 1.64 | 0.59 | 0.26 | 1.32 | 0.55 | 0.27 | 1.12 | 1.04 | 0.73 | 1.48 | 0.88 | 0.62 | 1.24 |
|  | 2 | 1.05 | 0.74 | 1.50 | 0.96 | 0.65 | 1.42 | 1.26 | 0.66 | 2.44 | 1.10 | 0.62 | 1.94 | 0.96 | 0.70 | 1.33 | 1.17 | 0.87 | 1.58 |
| 5% | 0 | 1.30 | 0.99 | 1.71 | 1.18 | 0.88 | 1.56 | 1.38 | 0.87 | 2.18 | 1.37 | 0.88 | 2.15 | 1.22 | 0.95 | 1.57 | 1.11 | 0.88 | 1.39 |
|  | 1 | 0.99 | 0.73 | 1.34 | 1.05 | 0.77 | 1.43 | 0.82 | 0.50 | 1.35 | 0.78 | 0.47 | 1.29 | 0.98 | 0.75 | 1.29 | 1.03 | 0.80 | 1.33 |
|  | 2 | 1.09 | 0.83 | 1.44 | 0.98 | 0.74 | 1.31 | 1.32 | 0.85 | 2.05 | 1.10 | 0.71 | 1.69 | 1.01 | 0.79 | 1.30 | 1.14 | 0.91 | 1.43 |
| 10% | 0 | 1.29 | 1.00 | 1.67 | 1.15 | 0.87 | 1.51 | 1.52 | 1.01 | 2.27 | 1.25 | 0.86 | 1.83 | 1.26 | 1.00 | 1.59 | 1.07 | 0.87 | 1.32 |
|  | 1 | 1.02 | 0.77 | 1.34 | 1.03 | 0.76 | 1.38 | 0.98 | 0.64 | 1.49 | 1.12 | 0.75 | 1.67 | 0.95 | 0.74 | 1.22 | 1.13 | 0.89 | 1.42 |
|  | 2 | 1.12 | 0.87 | 1.45 | 1.00 | 0.76 | 1.31 | 1.33 | 0.89 | 1.99 | 1.09 | 0.75 | 1.60 | 1.04 | 0.83 | 1.31 | 1.11 | 0.90 | 1.37 |
| 90% | 0 | 1.27 | 0.92 | 1.77 | 1.10 | 0.81 | 1.49 | 1.40 | 0.89 | 2.22 | 0.79 | 0.48 | 1.29 | 1.23 | 0.93 | 1.64 | 0.95 | 0.74 | 1.22 |
|  | 1 | 0.95 | 0.65 | 1.40 | 1.00 | 0.69 | 1.43 | 1.02 | 0.61 | 1.73 | 1.60 | 0.89 | 2.86 | 0.91 | 0.66 | 1.25 | 1.18 | 0.88 | 1.58 |
|  | 2 | 0.92 | 0.66 | 1.27 | 0.86 | 0.63 | 1.17 | 1.31 | 0.84 | 2.06 | 0.82 | 0.50 | 1.36 | 0.95 | 0.72 | 1.26 | 1.00 | 0.78 | 1.29 |
| 95% | 0 | 1.33 | 0.91 | 1.94 | 1.17 | 0.82 | 1.68 | 1.34 | 0.80 | 2.26 | 0.78 | 0.44 | 1.40 | 1.25 | 0.90 | 1.74 | 0.93 | 0.68 | 1.25 |
|  | 1 | 0.93 | 0.60 | 1.46 | 0.96 | 0.62 | 1.47 | 1.01 | 0.56 | 1.85 | 1.60 | 0.80 | 3.18 | 1.01 | 0.69 | 1.46 | 1.19 | 0.84 | 1.70 |
|  | 2 | 0.86 | 0.59 | 1.26 | 0.91 | 0.63 | 1.30 | 1.33 | 0.80 | 2.21 | 0.75 | 0.41 | 1.36 | 0.90 | 0.64 | 1.25 | 0.96 | 0.71 | 1.30 |
| 99% | 0 | 1.47 | 0.86 | 2.52 | 1.33 | 0.80 | 2.21 | 1.21 | 0.61 | 2.43 | 0.78 | 0.36 | 1.69 | 1.28 | 0.80 | 2.05 | 0.89 | 0.59 | 1.35 |
|  | 1 | 0.89 | 0.47 | 1.68 | 0.88 | 0.48 | 1.61 | 1.00 | 0.45 | 2.21 | 1.57 | 0.64 | 3.85 | 1.29 | 0.76 | 2.19 | 1.21 | 0.75 | 1.97 |
|  | 2 | 0.73 | 0.42 | 1.28 | 1.02 | 0.61 | 1.70 | 1.36 | 0.69 | 2.67 | 0.65 | 0.29 | 1.42 | 0.78 | 0.48 | 1.26 | 0.88 | 0.58 | 1.35 |
| Difficulty Sleeping | | | | | | | | | | | | | | | | | | | |
| 1% | 0 | 0.88 | 0.69 | 1.12 | 1.22 | 0.84 | 1.78 | 1.01 | 0.69 | 1.48 | 1.04 | 0.70 | 1.57 | 0.98 | 0.77 | 1.24 | 1.13 | 0.91 | 1.40 |
|  | 1 | 1.02 | 0.79 | 1.31 | 1.09 | 0.72 | 1.64 | 0.76 | 0.50 | 1.14 | 1.25 | 0.83 | 1.88 | 1.06 | 0.82 | 1.38 | 0.99 | 0.78 | 1.25 |
|  | 2 | 1.20 | 0.97 | 1.48 | 0.96 | 0.65 | 1.42 | 1.09 | 0.77 | 1.54 | 0.85 | 0.59 | 1.25 | 0.97 | 0.77 | 1.22 | 0.91 | 0.73 | 1.13 |
| 5% | 0 | 0.90 | 0.75 | 1.08 | 1.18 | 0.88 | 1.56 | 1.04 | 0.81 | 1.34 | 1.09 | 0.80 | 1.48 | 0.98 | 0.82 | 1.17 | 1.10 | 0.94 | 1.29 |
|  | 1 | 1.08 | 0.88 | 1.31 | 1.05 | 0.77 | 1.43 | 0.85 | 0.65 | 1.11 | 1.17 | 0.86 | 1.60 | 1.05 | 0.86 | 1.28 | 1.01 | 0.84 | 1.20 |
|  | 2 | 1.04 | 0.87 | 1.24 | 0.98 | 0.74 | 1.31 | 1.07 | 0.85 | 1.36 | 0.82 | 0.62 | 1.09 | 1.00 | 0.84 | 1.19 | 0.89 | 0.76 | 1.05 |
| 10% | 0 | 0.93 | 0.78 | 1.09 | 1.15 | 0.87 | 1.51 | 1.06 | 0.85 | 1.32 | 1.14 | 0.88 | 1.47 | 0.99 | 0.84 | 1.16 | 1.08 | 0.94 | 1.26 |
|  | 1 | 1.11 | 0.93 | 1.33 | 1.03 | 0.76 | 1.38 | 0.91 | 0.72 | 1.15 | 1.09 | 0.83 | 1.42 | 1.04 | 0.87 | 1.25 | 1.02 | 0.87 | 1.19 |
|  | 2 | 0.94 | 0.79 | 1.11 | 1.00 | 0.76 | 1.31 | 1.06 | 0.85 | 1.32 | 0.80 | 0.62 | 1.03 | 1.02 | 0.87 | 1.20 | 0.89 | 0.76 | 1.03 |
| 90% | 0 | 0.93 | 0.76 | 1.13 | 1.10 | 0.81 | 1.49 | 1.02 | 0.80 | 1.29 | 0.99 | 0.73 | 1.34 | 1.02 | 0.84 | 1.23 | 0.83 | 0.70 | 0.98 |
|  | 1 | 1.20 | 0.95 | 1.52 | 1.00 | 0.69 | 1.43 | 1.04 | 0.78 | 1.39 | 1.27 | 0.88 | 1.82 | 1.02 | 0.82 | 1.27 | 1.25 | 1.03 | 1.53 |
|  | 2 | 0.87 | 0.72 | 1.06 | 0.86 | 0.63 | 1.17 | 0.85 | 0.66 | 1.08 | 0.72 | 0.53 | 0.98 | 0.97 | 0.80 | 1.18 | 0.95 | 0.80 | 1.12 |
| 95% | 0 | 0.94 | 0.75 | 1.18 | 1.17 | 0.82 | 1.68 | 1.05 | 0.79 | 1.38 | 1.10 | 0.77 | 1.56 | 1.04 | 0.83 | 1.30 | 0.82 | 0.67 | 1.00 |
|  | 1 | 1.18 | 0.90 | 1.54 | 0.96 | 0.62 | 1.47 | 0.99 | 0.70 | 1.38 | 1.22 | 0.80 | 1.87 | 1.02 | 0.79 | 1.32 | 1.31 | 1.04 | 1.66 |
|  | 2 | 0.87 | 0.69 | 1.09 | 0.91 | 0.63 | 1.30 | 0.83 | 0.63 | 1.11 | 0.68 | 0.48 | 0.99 | 0.95 | 0.76 | 1.19 | 0.91 | 0.75 | 1.12 |
| 99% | 0 | 0.97 | 0.70 | 1.33 | 1.33 | 0.80 | 2.21 | 1.11 | 0.76 | 1.62 | 1.32 | 0.83 | 2.10 | 1.08 | 0.79 | 1.49 | 0.80 | 0.60 | 1.06 |
|  | 1 | 1.11 | 0.76 | 1.61 | 0.88 | 0.48 | 1.61 | 0.88 | 0.55 | 1.39 | 1.14 | 0.65 | 2.00 | 1.02 | 0.71 | 1.46 | 1.43 | 1.03 | 1.98 |
|  | 2 | 0.86 | 0.62 | 1.18 | 1.02 | 0.61 | 1.70 | 0.81 | 0.55 | 1.20 | 0.63 | 0.39 | 1.04 | 0.91 | 0.66 | 1.25 | 0.85 | 0.64 | 1.13 |
| Expressed Intent to Die | | | | | | | | | | | | | | | | | | | |
| 1% | 0 | 1.12 | 0.93 | 1.36 | 0.95 | 0.75 | 1.20 | 1.02 | 0.76 | 1.38 | 1.10 | 0.80 | 1.50 | 1.08 | 0.88 | 1.32 | 0.96 | 0.81 | 1.15 |
|  | 1 | 1.06 | 0.86 | 1.31 | 0.93 | 0.72 | 1.19 | 0.84 | 0.61 | 1.16 | 0.96 | 0.69 | 1.34 | 1.03 | 0.83 | 1.29 | 0.98 | 0.81 | 1.19 |
|  | 2 | 0.92 | 0.75 | 1.11 | 0.90 | 0.72 | 1.12 | 1.36 | 1.04 | 1.79 | 0.97 | 0.73 | 1.29 | 1.04 | 0.86 | 1.26 | 0.97 | 0.82 | 1.16 |
| 5% | 0 | 1.04 | 0.89 | 1.20 | 1.04 | 0.88 | 1.23 | 0.96 | 0.79 | 1.18 | 1.07 | 0.85 | 1.35 | 1.08 | 0.93 | 1.25 | 0.98 | 0.86 | 1.12 |
|  | 1 | 1.05 | 0.89 | 1.24 | 0.95 | 0.79 | 1.14 | 0.91 | 0.73 | 1.12 | 1.06 | 0.83 | 1.36 | 0.96 | 0.82 | 1.12 | 1.02 | 0.89 | 1.18 |
|  | 2 | 0.94 | 0.80 | 1.09 | 0.93 | 0.78 | 1.09 | 1.26 | 1.03 | 1.52 | 0.99 | 0.80 | 1.23 | 1.00 | 0.86 | 1.15 | 0.95 | 0.84 | 1.08 |
| 10% | 0 | 0.97 | 0.84 | 1.11 | 1.09 | 0.94 | 1.28 | 0.94 | 0.78 | 1.13 | 1.05 | 0.86 | 1.28 | 1.07 | 0.94 | 1.23 | 0.99 | 0.88 | 1.11 |
|  | 1 | 1.04 | 0.89 | 1.20 | 0.97 | 0.82 | 1.14 | 0.95 | 0.78 | 1.15 | 1.16 | 0.94 | 1.43 | 0.92 | 0.79 | 1.07 | 1.04 | 0.92 | 1.19 |
|  | 2 | 0.95 | 0.83 | 1.10 | 0.95 | 0.81 | 1.11 | 1.19 | 0.99 | 1.43 | 1.01 | 0.83 | 1.23 | 0.98 | 0.85 | 1.12 | 0.94 | 0.84 | 1.06 |
| 90% | 0 | 0.98 | 0.82 | 1.16 | 1.10 | 0.93 | 1.30 | 0.88 | 0.72 | 1.08 | 1.04 | 0.82 | 1.32 | 1.09 | 0.94 | 1.27 | 0.95 | 0.83 | 1.09 |
|  | 1 | 1.02 | 0.83 | 1.25 | 1.09 | 0.90 | 1.33 | 1.14 | 0.90 | 1.44 | 1.02 | 0.78 | 1.34 | 1.00 | 0.84 | 1.19 | 1.08 | 0.92 | 1.26 |
|  | 2 | 0.98 | 0.83 | 1.17 | 0.89 | 0.75 | 1.05 | 0.95 | 0.77 | 1.17 | 1.02 | 0.81 | 1.30 | 0.90 | 0.77 | 1.05 | 0.98 | 0.86 | 1.13 |
| 95% | 0 | 0.97 | 0.80 | 1.19 | 1.13 | 0.93 | 1.38 | 0.90 | 0.72 | 1.13 | 1.06 | 0.81 | 1.40 | 1.13 | 0.94 | 1.34 | 0.92 | 0.78 | 1.08 |
|  | 1 | 1.03 | 0.82 | 1.30 | 1.11 | 0.88 | 1.40 | 1.16 | 0.88 | 1.53 | 0.94 | 0.68 | 1.30 | 1.01 | 0.83 | 1.25 | 1.10 | 0.91 | 1.33 |
|  | 2 | 0.98 | 0.81 | 1.20 | 0.86 | 0.70 | 1.05 | 0.90 | 0.71 | 1.14 | 1.02 | 0.77 | 1.35 | 0.88 | 0.73 | 1.05 | 0.98 | 0.83 | 1.15 |
| 99% | 0 | 0.97 | 0.73 | 1.28 | 1.18 | 0.89 | 1.56 | 0.95 | 0.69 | 1.31 | 1.10 | 0.76 | 1.59 | 1.21 | 0.94 | 1.56 | 0.87 | 0.69 | 1.10 |
|  | 1 | 1.05 | 0.76 | 1.45 | 1.15 | 0.84 | 1.59 | 1.20 | 0.82 | 1.76 | 0.81 | 0.52 | 1.26 | 1.06 | 0.79 | 1.42 | 1.14 | 0.88 | 1.49 |
|  | 2 | 0.98 | 0.74 | 1.30 | 0.82 | 0.61 | 1.09 | 0.80 | 0.57 | 1.11 | 1.00 | 0.69 | 1.47 | 0.82 | 0.63 | 1.06 | 0.97 | 0.77 | 1.22 |
| Little to No Engagement with Crisis Counselor | | | | | | | | | | | | | | | | | | | |
| 1% | 0 | 1.62 | 0.99 | 2.63 | 0.87 | 0.49 | 1.57 | 1.29 | 0.64 | 2.57 | 1.01 | 0.44 | 2.35 | 1.06 | 0.71 | 1.60 | 0.97 | 0.66 | 1.44 |
|  | 1 | 0.62 | 0.34 | 1.15 | 1.32 | 0.71 | 2.46 | 0.67 | 0.27 | 1.66 | 1.32 | 0.54 | 3.22 | 1.13 | 0.72 | 1.78 | 0.64 | 0.39 | 1.03 |
|  | 2 | 0.93 | 0.55 | 1.56 | 0.47 | 0.24 | 0.92 | 0.60 | 0.27 | 1.33 | 0.63 | 0.27 | 1.48 | 0.67 | 0.43 | 1.03 | 0.98 | 0.65 | 1.47 |
| 5% | 0 | 1.37 | 0.93 | 2.02 | 0.87 | 0.56 | 1.36 | 1.14 | 0.69 | 1.88 | 0.90 | 0.48 | 1.67 | 1.02 | 0.75 | 1.39 | 0.92 | 0.69 | 1.23 |
|  | 1 | 0.75 | 0.48 | 1.18 | 1.27 | 0.79 | 2.07 | 0.89 | 0.49 | 1.63 | 1.52 | 0.79 | 2.92 | 1.02 | 0.73 | 1.44 | 0.87 | 0.62 | 1.21 |
|  | 2 | 0.84 | 0.56 | 1.25 | 0.61 | 0.38 | 0.98 | 0.57 | 0.33 | 0.99 | 0.65 | 0.35 | 1.21 | 0.77 | 0.56 | 1.06 | 1.06 | 0.79 | 1.41 |
| 10% | 0 | 1.21 | 0.84 | 1.75 | 0.88 | 0.58 | 1.32 | 1.05 | 0.67 | 1.65 | 0.81 | 0.47 | 1.38 | 0.99 | 0.75 | 1.31 | 0.90 | 0.69 | 1.17 |
|  | 1 | 0.86 | 0.57 | 1.28 | 1.24 | 0.79 | 1.93 | 1.07 | 0.66 | 1.74 | 1.71 | 0.99 | 2.96 | 0.94 | 0.69 | 1.28 | 1.04 | 0.78 | 1.39 |
|  | 2 | 0.80 | 0.55 | 1.16 | 0.72 | 0.47 | 1.09 | 0.57 | 0.36 | 0.91 | 0.69 | 0.40 | 1.17 | 0.86 | 0.65 | 1.14 | 1.10 | 0.85 | 1.44 |
| 90% | 0 | 1.04 | 0.65 | 1.65 | 0.84 | 0.53 | 1.35 | 0.93 | 0.58 | 1.50 | 0.89 | 0.41 | 1.92 | 1.03 | 0.76 | 1.40 | 0.94 | 0.68 | 1.29 |
|  | 1 | 0.90 | 0.52 | 1.55 | 1.14 | 0.66 | 1.96 | 0.78 | 0.44 | 1.40 | 2.05 | 0.82 | 5.11 | 1.00 | 0.69 | 1.44 | 0.99 | 0.68 | 1.44 |
|  | 2 | 0.97 | 0.62 | 1.51 | 1.07 | 0.67 | 1.70 | 1.06 | 0.66 | 1.69 | 0.31 | 0.14 | 0.69 | 1.00 | 0.74 | 1.36 | 1.14 | 0.83 | 1.56 |
| 95% | 0 | 1.09 | 0.65 | 1.83 | 0.88 | 0.52 | 1.50 | 0.91 | 0.52 | 1.57 | 1.03 | 0.42 | 2.51 | 1.10 | 0.77 | 1.57 | 0.93 | 0.64 | 1.35 |
|  | 1 | 0.83 | 0.45 | 1.53 | 1.15 | 0.62 | 2.13 | 0.65 | 0.33 | 1.29 | 2.45 | 0.86 | 7.03 | 0.90 | 0.59 | 1.39 | 0.91 | 0.59 | 1.42 |
|  | 2 | 1.05 | 0.63 | 1.74 | 1.12 | 0.66 | 1.89 | 1.01 | 0.59 | 1.73 | 0.26 | 0.10 | 0.67 | 1.03 | 0.72 | 1.47 | 1.22 | 0.84 | 1.76 |
| 99% | 0 | 1.21 | 0.59 | 2.47 | 0.98 | 0.48 | 2.04 | 0.86 | 0.40 | 1.86 | 1.29 | 0.41 | 4.00 | 1.26 | 0.76 | 2.08 | 0.92 | 0.54 | 1.56 |
|  | 1 | 0.67 | 0.29 | 1.55 | 1.15 | 0.50 | 2.65 | 0.44 | 0.17 | 1.15 | 3.14 | 0.85 | 11.59 | 0.72 | 0.40 | 1.32 | 0.77 | 0.41 | 1.43 |
|  | 2 | 1.29 | 0.65 | 2.57 | 1.23 | 0.60 | 2.52 | 0.91 | 0.43 | 1.95 | 0.21 | 0.06 | 0.71 | 1.08 | 0.66 | 1.79 | 1.39 | 0.83 | 2.31 |
| Few Future Plans | | | | | | | | | | | | | | | | | | | |
| 1% | 0 | 1.55 | 1.13 | 2.11 | 1.17 | 0.82 | 1.66 | 1.28 | 0.81 | 2.01 | 1.65 | 0.99 | 2.76 | 1.49 | 1.11 | 2.00 | 0.99 | 0.74 | 1.32 |
|  | 1 | 1.00 | 0.68 | 1.46 | 1.02 | 0.69 | 1.53 | 0.86 | 0.51 | 1.44 | 0.69 | 0.38 | 1.26 | 0.70 | 0.50 | 0.99 | 0.86 | 0.61 | 1.19 |
|  | 2 | 0.85 | 0.61 | 1.19 | 1.04 | 0.72 | 1.51 | 1.64 | 1.09 | 2.48 | 0.79 | 0.47 | 1.34 | 1.35 | 1.01 | 1.81 | 1.06 | 0.79 | 1.42 |
| 5% | 0 | 1.27 | 0.98 | 1.64 | 1.06 | 0.80 | 1.40 | 1.08 | 0.78 | 1.50 | 1.30 | 0.89 | 1.91 | 1.33 | 1.06 | 1.67 | 0.95 | 0.77 | 1.19 |
|  | 1 | 1.11 | 0.82 | 1.49 | 0.98 | 0.72 | 1.33 | 1.02 | 0.72 | 1.46 | 0.82 | 0.53 | 1.27 | 0.73 | 0.56 | 0.95 | 0.91 | 0.72 | 1.16 |
|  | 2 | 0.81 | 0.62 | 1.06 | 1.03 | 0.78 | 1.37 | 1.27 | 0.93 | 1.74 | 0.89 | 0.60 | 1.31 | 1.24 | 0.99 | 1.56 | 0.98 | 0.79 | 1.21 |
| 10% | 0 | 1.08 | 0.85 | 1.38 | 1.01 | 0.77 | 1.32 | 1.00 | 0.73 | 1.35 | 1.03 | 0.73 | 1.44 | 1.21 | 0.98 | 1.50 | 0.94 | 0.77 | 1.15 |
|  | 1 | 1.19 | 0.91 | 1.55 | 0.96 | 0.72 | 1.28 | 1.11 | 0.81 | 1.53 | 0.98 | 0.68 | 1.39 | 0.75 | 0.60 | 0.96 | 0.95 | 0.76 | 1.18 |
|  | 2 | 0.80 | 0.62 | 1.02 | 1.03 | 0.78 | 1.34 | 1.10 | 0.81 | 1.49 | 1.00 | 0.71 | 1.41 | 1.16 | 0.93 | 1.43 | 0.94 | 0.77 | 1.14 |
| 90% | 0 | 0.96 | 0.72 | 1.28 | 1.06 | 0.80 | 1.40 | 1.11 | 0.80 | 1.53 | 1.23 | 0.81 | 1.87 | 1.34 | 1.05 | 1.71 | 0.88 | 0.70 | 1.11 |
|  | 1 | 1.10 | 0.78 | 1.55 | 0.92 | 0.66 | 1.29 | 1.09 | 0.74 | 1.60 | 1.09 | 0.66 | 1.79 | 0.83 | 0.62 | 1.10 | 1.19 | 0.91 | 1.55 |
|  | 2 | 1.22 | 0.92 | 1.62 | 1.17 | 0.87 | 1.55 | 0.95 | 0.69 | 1.32 | 0.84 | 0.54 | 1.28 | 1.18 | 0.93 | 1.51 | 1.02 | 0.81 | 1.29 |
| 95% | 0 | 1.03 | 0.74 | 1.42 | 1.16 | 0.84 | 1.61 | 1.18 | 0.82 | 1.70 | 1.48 | 0.92 | 2.37 | 1.49 | 1.14 | 1.96 | 0.91 | 0.70 | 1.19 |
|  | 1 | 1.07 | 0.73 | 1.56 | 0.89 | 0.60 | 1.32 | 1.04 | 0.67 | 1.63 | 1.02 | 0.57 | 1.82 | 0.80 | 0.58 | 1.11 | 1.25 | 0.92 | 1.71 |
|  | 2 | 1.32 | 0.96 | 1.81 | 1.21 | 0.86 | 1.69 | 0.95 | 0.66 | 1.38 | 0.90 | 0.55 | 1.47 | 1.21 | 0.92 | 1.59 | 1.03 | 0.78 | 1.34 |
| 99% | 0 | 1.23 | 0.78 | 1.92 | 1.41 | 0.90 | 2.22 | 1.35 | 0.82 | 2.23 | 1.99 | 1.09 | 3.61 | 1.95 | 1.33 | 2.85 | 0.99 | 0.68 | 1.43 |
|  | 1 | 0.98 | 0.58 | 1.66 | 0.83 | 0.49 | 1.43 | 0.94 | 0.51 | 1.73 | 0.92 | 0.44 | 1.93 | 0.74 | 0.47 | 1.16 | 1.40 | 0.91 | 2.15 |
|  | 2 | 1.61 | 1.04 | 2.50 | 1.28 | 0.80 | 2.05 | 0.96 | 0.57 | 1.59 | 1.03 | 0.55 | 1.93 | 1.26 | 0.85 | 1.86 | 1.03 | 0.71 | 1.49 |
