# Supplementary material for: Temperature extremes contribute to suicide-related help-seeking through multiple pathways: Evidence from crisis hotline data (2019–2023)
Source: PLOS Ment Health. 2026 Feb 11;3(2):e0000501. doi: 10.1371/journal.pmen.0000501 (PMC12893560; doi:10.1371/journal.pmen.0000501)
Supplement: S3 Table — (DOCX) [file pmen.0000501.s005.docx]

S3 Table. Lagged (days 0–10) DLNM results; estimates are derived compared to median minimum temperature.

| **Percentile** | **Lag** | **Suicide Calls** | | **Availability of Means** | | **Difficulty Sleeping** | | **Expressed Intent** | | **Difficulty Engaging** | | **Few Future Plans** | |
| --- | --- | --- | --- | --- | --- | --- | --- | --- | --- | --- | --- | --- | --- |
|  |  | **RR** | **CI** | **RR** | **CI** | **RR** | **CI** | **RR** | **CI** | **RR** | **CI** | **RR** | **CI** |
| 1% | 0 | 0.88 | 0.81-0.96 | 1.05 | 0.93-1.19 | 0.97 | 0.86-1.08 | 0.95 | 0.86-1.05 | 0.98 | 0.85-1.14 | 1.03 | 0.91-1.17 |
| 1% | 1 | 0.91 | 0.86-0.96 | 1.04 | 0.96-1.12 | 0.96 | 0.90-1.03 | 0.96 | 0.91-1.02 | 0.97 | 0.89-1.06 | 1.01 | 0.94-1.09 |
| 1% | 2 | 0.93 | 0.90-0.97 | 1.02 | 0.97-1.08 | 0.96 | 0.92-1.00 | 0.97 | 0.93-1.01 | 0.96 | 0.90-1.02 | 0.99 | 0.94-1.04 |
| 1% | 3 | 0.95 | 0.92-0.99 | 1.01 | 0.95-1.07 | 0.95 | 0.91-1.00 | 0.97 | 0.93-1.02 | 0.95 | 0.88-1.02 | 0.97 | 0.92-1.03 |
| 1% | 4 | 0.96 | 0.92-1.01 | 1.00 | 0.93-1.06 | 0.95 | 0.90-1.01 | 0.97 | 0.92-1.03 | 0.94 | 0.86-1.02 | 0.96 | 0.90-1.03 |
| 1% | 5 | 0.96 | 0.91-1.00 | 0.98 | 0.92-1.05 | 0.95 | 0.89-1.00 | 0.97 | 0.92-1.02 | 0.93 | 0.86-1.01 | 0.95 | 0.89-1.02 |
| 1% | 6 | 0.95 | 0.91-0.99 | 0.97 | 0.91-1.02 | 0.94 | 0.89-0.99 | 0.96 | 0.92-1.00 | 0.92 | 0.86-0.99 | 0.95 | 0.89-1.00 |
| 1% | 7 | 0.93 | 0.90-0.96 | 0.95 | 0.90-1.00 | 0.94 | 0.90-0.98 | 0.94 | 0.91-0.98 | 0.92 | 0.86-0.97 | 0.94 | 0.90-0.99 |
| 1% | 8 | 0.91 | 0.87-0.94 | 0.93 | 0.88-0.99 | 0.93 | 0.89-0.98 | 0.93 | 0.89-0.97 | 0.91 | 0.85-0.97 | 0.94 | 0.89-1.00 |
| 1% | 9 | 0.88 | 0.83-0.93 | 0.92 | 0.84-1.00 | 0.93 | 0.86-1.00 | 0.91 | 0.86-0.97 | 0.90 | 0.82-1.00 | 0.94 | 0.87-1.03 |
| 1% | 10 | 0.86 | 0.79-0.93 | 0.90 | 0.80-1.02 | 0.92 | 0.83-1.02 | 0.90 | 0.81-0.99 | 0.90 | 0.78-1.04 | 0.95 | 0.84-1.07 |
| 5% | 0 | 0.91 | 0.86-0.97 | 1.06 | 0.97-1.16 | 0.99 | 0.91-1.07 | 0.96 | 0.90-1.03 | 0.93 | 0.83-1.03 | 1.01 | 0.92-1.10 |
| 5% | 1 | 0.93 | 0.90-0.97 | 1.04 | 0.99-1.10 | 0.98 | 0.93-1.02 | 0.97 | 0.93-1.01 | 0.94 | 0.88-1.00 | 0.99 | 0.94-1.05 |
| 5% | 2 | 0.95 | 0.93-0.97 | 1.02 | 0.99-1.06 | 0.96 | 0.93-1.00 | 0.98 | 0.95-1.00 | 0.95 | 0.91-0.99 | 0.98 | 0.95-1.01 |
| 5% | 3 | 0.96 | 0.94-0.99 | 1.01 | 0.97-1.05 | 0.96 | 0.92-0.99 | 0.98 | 0.95-1.01 | 0.96 | 0.92-1.01 | 0.97 | 0.93-1.01 |
| 5% | 4 | 0.97 | 0.94-1.00 | 0.99 | 0.95-1.04 | 0.95 | 0.91-0.99 | 0.98 | 0.95-1.02 | 0.97 | 0.91-1.02 | 0.96 | 0.92-1.01 |
| 5% | 5 | 0.97 | 0.94-1.01 | 0.98 | 0.94-1.03 | 0.95 | 0.91-0.99 | 0.98 | 0.95-1.02 | 0.97 | 0.91-1.02 | 0.96 | 0.92-1.01 |
| 5% | 6 | 0.97 | 0.95-1.00 | 0.98 | 0.94-1.02 | 0.95 | 0.92-0.99 | 0.98 | 0.95-1.01 | 0.97 | 0.92-1.01 | 0.96 | 0.92-1.00 |
| 5% | 7 | 0.97 | 0.94-0.99 | 0.97 | 0.94-1.00 | 0.96 | 0.93-0.99 | 0.97 | 0.95-1.00 | 0.96 | 0.92-1.00 | 0.96 | 0.93-1.00 |
| 5% | 8 | 0.96 | 0.93-0.98 | 0.96 | 0.93-1.00 | 0.97 | 0.94-1.00 | 0.97 | 0.94-1.00 | 0.96 | 0.92-1.00 | 0.97 | 0.93-1.01 |
| 5% | 9 | 0.95 | 0.91-0.98 | 0.96 | 0.91-1.02 | 0.98 | 0.93-1.03 | 0.96 | 0.92-1.00 | 0.95 | 0.89-1.01 | 0.97 | 0.92-1.03 |
| 5% | 10 | 0.94 | 0.88-0.99 | 0.96 | 0.88-1.04 | 0.99 | 0.92-1.06 | 0.95 | 0.89-1.02 | 0.94 | 0.86-1.04 | 0.98 | 0.90-1.07 |
| 10% | 0 | 0.94 | 0.89-0.99 | 1.07 | 0.98-1.16 | 1.00 | 0.93-1.08 | 0.97 | 0.91-1.04 | 0.90 | 0.82-0.99 | 1.00 | 0.92-1.08 |
| 10% | 1 | 0.95 | 0.92-0.98 | 1.04 | 0.99-1.10 | 0.99 | 0.94-1.03 | 0.98 | 0.94-1.02 | 0.93 | 0.87-0.98 | 0.99 | 0.94-1.04 |
| 10% | 2 | 0.96 | 0.94-0.98 | 1.02 | 0.99-1.06 | 0.97 | 0.94-1.00 | 0.98 | 0.96-1.01 | 0.95 | 0.91-0.99 | 0.98 | 0.94-1.01 |
| 10% | 3 | 0.97 | 0.95-1.00 | 1.01 | 0.97-1.05 | 0.96 | 0.93-0.99 | 0.99 | 0.96-1.01 | 0.97 | 0.93-1.01 | 0.97 | 0.93-1.01 |
| 10% | 4 | 0.98 | 0.95-1.01 | 0.99 | 0.95-1.04 | 0.96 | 0.92-0.99 | 0.99 | 0.95-1.02 | 0.98 | 0.94-1.04 | 0.97 | 0.93-1.01 |
| 10% | 5 | 0.99 | 0.96-1.01 | 0.99 | 0.94-1.03 | 0.96 | 0.92-1.00 | 0.99 | 0.96-1.02 | 0.99 | 0.94-1.04 | 0.97 | 0.93-1.01 |
| 10% | 6 | 0.99 | 0.96-1.01 | 0.98 | 0.94-1.02 | 0.97 | 0.93-1.00 | 0.99 | 0.96-1.02 | 0.99 | 0.95-1.04 | 0.97 | 0.93-1.01 |
| 10% | 7 | 0.99 | 0.97-1.01 | 0.98 | 0.95-1.01 | 0.98 | 0.95-1.00 | 0.99 | 0.97-1.02 | 0.99 | 0.96-1.03 | 0.98 | 0.95-1.01 |
| 10% | 8 | 0.99 | 0.97-1.01 | 0.98 | 0.95-1.02 | 0.99 | 0.96-1.02 | 0.99 | 0.96-1.02 | 0.99 | 0.95-1.03 | 0.98 | 0.95-1.02 |
| 10% | 9 | 0.99 | 0.96-1.02 | 0.98 | 0.93-1.04 | 1.01 | 0.96-1.05 | 0.99 | 0.95-1.03 | 0.98 | 0.92-1.04 | 0.99 | 0.94-1.04 |
| 10% | 10 | 0.99 | 0.94-1.04 | 0.99 | 0.91-1.07 | 1.03 | 0.96-1.09 | 0.99 | 0.93-1.05 | 0.97 | 0.89-1.06 | 1.00 | 0.93-1.08 |
| 90% | 0 | 1.10 | **1.03-1.18** | 1.08 | 0.97-1.20 | 1.10 | **1.00-1.20** | 1.08 | **1.00-1.17** | 1.09 | 0.96-1.23 | 1.08 | 0.97-1.20 |
| 90% | 1 | 1.06 | **1.02-1.10** | 1.06 | 0.99-1.13 | 1.06 | **1.00-1.12** | 1.05 | **1.00-1.11** | 1.05 | 0.98-1.13 | 1.04 | 0.98-1.11 |
| 90% | 2 | 1.02 | **1.00-1.05** | 1.04 | 0.99-1.08 | 1.02 | 0.99-1.06 | 1.03 | **1.00-1.06** | 1.02 | 0.97-1.06 | 1.01 | 0.97-1.05 |
| 90% | 3 | 1.00 | 0.97-1.03 | 1.02 | 0.97-1.07 | 1.00 | 0.96-1.04 | 1.01 | 0.98-1.05 | 0.99 | 0.94-1.04 | 0.99 | 0.95-1.04 |
| 90% | 4 | 0.98 | 0.95-1.02 | 1.01 | 0.95-1.07 | 0.98 | 0.93-1.03 | 1.00 | 0.96-1.04 | 0.98 | 0.91-1.04 | 0.98 | 0.93-1.04 |
| 90% | 5 | 0.98 | 0.94-1.01 | 1.00 | 0.94-1.06 | 0.98 | 0.93-1.02 | 0.99 | 0.95-1.04 | 0.97 | 0.91-1.03 | 0.98 | 0.93-1.04 |
| 90% | 6 | 0.98 | 0.95-1.01 | 1.00 | 0.95-1.05 | 0.98 | 0.94-1.02 | 0.99 | 0.96-1.03 | 0.97 | 0.92-1.03 | 0.99 | 0.94-1.04 |
| 90% | 7 | 0.99 | 0.97-1.02 | 1.00 | 0.96-1.04 | 0.99 | 0.96-1.02 | 1.00 | 0.97-1.03 | 0.98 | 0.94-1.02 | 1.00 | 0.97-1.04 |
| 90% | 8 | 1.01 | 0.98-1.03 | 1.00 | 0.96-1.04 | 1.00 | 0.97-1.04 | 1.00 | 0.97-1.04 | 0.99 | 0.95-1.04 | 1.02 | 0.98-1.06 |
| 90% | 9 | 1.03 | 0.99-1.07 | 1.01 | 0.94-1.07 | 1.02 | 0.97-1.08 | 1.01 | 0.97-1.06 | 1.01 | 0.94-1.08 | 1.05 | 0.98-1.11 |
| 90% | 10 | 1.05 | 0.99-1.11 | 1.01 | 0.92-1.12 | 1.04 | 0.96-1.13 | 1.02 | 0.95-1.10 | 1.03 | 0.92-1.14 | 1.07 | 0.98-1.18 |
| 95% | 0 | 1.17 | **1.08-1.26** | 1.13 | **1.00-1.28** | 1.17 | **1.06-1.30** | 1.14 | **1.04-1.25** | 1.17 | **1.03-1.34** | 1.16 | **1.03-1.30** |
| 95% | 1 | 1.11 | **1.06-1.16** | 1.10 | **1.02-1.18** | 1.11 | **1.04-1.18** | 1.10 | **1.04-1.16** | 1.11 | **1.02-1.20** | 1.10 | **1.03-1.18** |
| 95% | 2 | 1.06 | **1.03-1.09** | 1.07 | **1.02-1.11** | 1.05 | **1.02-1.09** | 1.06 | **1.03-1.10** | 1.05 | **1.00-1.10** | 1.05 | **1.01-1.09** |
| 95% | 3 | 1.02 | 0.98-1.05 | 1.04 | 0.99-1.10 | 1.02 | 0.97-1.06 | 1.03 | 0.99-1.07 | 1.01 | 0.95-1.07 | 1.01 | 0.96-1.07 |
| 95% | 4 | 1.00 | 0.96-1.04 | 1.03 | 0.96-1.10 | 0.99 | 0.94-1.05 | 1.01 | 0.97-1.07 | 0.98 | 0.91-1.06 | 0.99 | 0.93-1.06 |
| 95% | 5 | 0.99 | 0.95-1.03 | 1.02 | 0.95-1.09 | 0.98 | 0.93-1.04 | 1.01 | 0.96-1.06 | 0.97 | 0.91-1.05 | 0.99 | 0.93-1.06 |
| 95% | 6 | 0.99 | 0.96-1.03 | 1.01 | 0.96-1.07 | 0.99 | 0.94-1.03 | 1.01 | 0.96-1.05 | 0.98 | 0.92-1.04 | 1.00 | 0.95-1.06 |
| 95% | 7 | 1.01 | 0.98-1.04 | 1.01 | 0.97-1.06 | 1.00 | 0.96-1.04 | 1.01 | 0.98-1.05 | 0.99 | 0.95-1.04 | 1.02 | 0.98-1.07 |
| 95% | 8 | 1.03 | **1.00-1.06** | 1.02 | 0.97-1.06 | 1.02 | 0.98-1.06 | 1.02 | 0.99-1.06 | 1.02 | 0.97-1.07 | 1.05 | **1.01-1.10** |
| 95% | 9 | 1.06 | **1.01-1.11** | 1.02 | 0.95-1.10 | 1.05 | 0.99-1.11 | 1.04 | 0.99-1.10 | 1.04 | 0.97-1.13 | 1.09 | **1.02-1.16** |
| 95% | 10 | 1.09 | **1.02-1.16** | 1.03 | 0.92-1.15 | 1.07 | 0.98-1.18 | 1.06 | 0.97-1.15 | 1.08 | 0.96-1.21 | 1.13 | **1.02-1.25** |
| 99% | 0 | 1.31 | **1.19-1.44** | 1.24 | **1.05-1.46** | 1.33 | **1.16-1.52** | 1.27 | **1.13-1.44** | 1.37 | **1.15-1.63** | 1.33 | **1.15-1.54** |
| 99% | 1 | 1.21 | **1.14-1.28** | 1.18 | **1.07-1.29** | 1.22 | **1.13-1.31** | 1.19 | **1.11-1.28** | 1.23 | **1.11-1.36** | 1.22 | **1.12-1.32** |
| 99% | 2 | 1.12 | **1.09-1.16** | 1.13 | **1.07-1.19** | 1.12 | **1.07-1.18** | 1.13 | **1.08-1.17** | 1.12 | **1.05-1.19** | 1.12 | **1.07-1.18** |
| 99% | 3 | 1.06 | **1.02-1.11** | 1.09 | **1.01-1.17** | 1.06 | **1.00-1.12** | 1.08 | **1.02-1.13** | 1.04 | 0.97-1.12 | 1.06 | 0.99-1.13 |
| 99% | 4 | 1.03 | 0.98-1.08 | 1.06 | 0.97-1.16 | 1.02 | 0.95-1.09 | 1.05 | 0.98-1.11 | 1.00 | 0.91-1.10 | 1.02 | 0.94-1.11 |
| 99% | 5 | 1.02 | 0.97-1.07 | 1.05 | 0.96-1.14 | 1.00 | 0.93-1.08 | 1.03 | 0.97-1.10 | 0.98 | 0.90-1.08 | 1.01 | 0.94-1.10 |
| 99% | 6 | 1.03 | 0.98-1.07 | 1.04 | 0.97-1.12 | 1.01 | 0.95-1.07 | 1.03 | 0.98-1.09 | 0.99 | 0.92-1.08 | 1.03 | 0.96-1.10 |
| 99% | 7 | 1.05 | **1.01-1.08** | 1.04 | 0.99-1.10 | 1.03 | 0.98-1.07 | 1.05 | **1.00-1.09** | 1.02 | 0.96-1.08 | 1.06 | **1.01-1.12** |
| 99% | 8 | 1.08 | **1.05-1.12** | 1.05 | 0.99-1.11 | 1.06 | **1.01-1.11** | 1.07 | **1.02-1.11** | 1.07 | **1.01-1.13** | 1.11 | **1.06-1.17** |
| 99% | 9 | 1.13 | **1.07-1.19** | 1.06 | 0.97-1.16 | 1.10 | **1.02-1.19** | 1.09 | **1.02-1.17** | 1.12 | **1.02-1.24** | 1.17 | **1.08-1.27** |
| 99% | 10 | 1.17 | **1.08-1.28** | 1.07 | 0.93-1.24 | 1.15 | **1.02-1.29** | 1.13 | **1.01-1.25** | 1.19 | **1.02-1.38** | 1.24 | **1.09-1.41** |
