# Supplementary material for: Temperature extremes contribute to suicide-related help-seeking through multiple pathways: Evidence from crisis hotline data (2019–2023)
Source: PLOS Ment Health. 2026 Feb 11;3(2):e0000501. doi: 10.1371/journal.pmen.0000501 (PMC12893560; doi:10.1371/journal.pmen.0000501)
Supplement: S4 Table — (DOCX) [file pmen.0000501.s006.docx]

S4 Table. Prevalence ratio point estimates for suicide-related crisis conversations at average temperature extremes (e.g., 5th, 95th); resulted reported in reference to median temperature. Overall estimates (days 0-2) reported.

| **Percentile** | **Average Temperature** | | | **Maximum Temperature** | | |
| --- | --- | --- | --- | --- | --- | --- |
|  | **Prevalence Ratio** | **Lower Confidence Interval** | **Upper Confidence Interval** | **Prevalence Ratio** | **Lower Confidence Interval** | **Upper Confidence Interval** |
| Suicide Calls | | | | | | |
| 1st | 0.45 | 0.38 | 0.54 | 0.50 | 0.42 | 0.61 |
| 5th | 0.61 | 0.55 | 0.68 | 0.69 | 0.62 | 0.77 |
| 10th | 0.72 | 0.65 | 0.80 | 0.79 | 0.71 | 0.87 |
| 90th | 1.08 | 1.00 | 1.17 | 0.89 | 0.82 | 0.97 |
| 95th | 1.18 | 1.09 | 1.28 | 0.79 | 0.72 | 0.87 |
| 99th | 1.49 | 1.32 | 1.67 | 0.55 | 0.47 | 0.65 |
| Means Available | | | | | | |
| 1st | 0.83 | 0.65 | 1.05 | 0.94 | 0.74 | 1.19 |
| 5th | 0.97 | 0.83 | 1.14 | 1.08 | 0.92 | 1.27 |
| 10th | 1.04 | 0.89 | 1.22 | 1.12 | 0.96 | 1.31 |
| 90th | 1.16 | 1.03 | 1.32 | 1.03 | 0.91 | 1.18 |
| 95th | 1.27 | 1.11 | 1.44 | 0.94 | 0.81 | 1.08 |
| 99th | 1.53 | 1.26 | 1.84 | 0.69 | 0.54 | 0.89 |
| Difficulty Sleeping | | | | | | |
| 1st | 0.59 | 0.47 | 0.74 | 0.67 | 0.54 | 0.84 |
| 5th | 0.72 | 0.63 | 0.83 | 0.79 | 0.69 | 0.91 |
| 10th | 0.80 | 0.71 | 0.92 | 0.86 | 0.75 | 0.98 |
| 90th | 1.04 | 0.94 | 1.15 | 0.88 | 0.79 | 0.98 |
| 95th | 1.14 | 1.02 | 1.27 | 0.80 | 0.71 | 0.90 |
| 99th | 1.43 | 1.22 | 1.68 | 0.60 | 0.49 | 0.74 |
| Expressed Intent to Die | | | | | | |
| 1st | 0.57 | 0.47 | 0.70 | 0.65 | 0.53 | 0.79 |
| 5th | 0.73 | 0.64 | 0.82 | 0.81 | 0.72 | 0.92 |
| 10th | 0.82 | 0.73 | 0.92 | 0.89 | 0.79 | 1.01 |
| 90th | 1.13 | 1.03 | 1.23 | 0.96 | 0.88 | 1.06 |
| 95th | 1.24 | 1.12 | 1.36 | 0.87 | 0.78 | 0.96 |
| 99th | 1.57 | 1.36 | 1.80 | 0.62 | 0.51 | 0.75 |
| Difficulty Engaging with Crisis Counselor | | | | | | |
| 1st | 0.67 | 0.51 | 0.87 | 0.75 | 0.57 | 0.99 |
| 5th | 0.71 | 0.59 | 0.85 | 0.80 | 0.66 | 0.95 |
| 10th | 0.75 | 0.63 | 0.89 | 0.83 | 0.70 | 0.99 |
| 90th | 1.03 | 0.90 | 1.18 | 0.93 | 0.81 | 1.07 |
| 95th | 1.14 | 1.00 | 1.32 | 0.87 | 0.75 | 1.01 |
| 99th | 1.47 | 1.21 | 1.80 | 0.73 | 0.57 | 0.94 |
| Few Future Plans | | | | | | |
| 1st | 0.69 | 0.55 | 0.88 | 0.77 | 0.60 | 0.98 |
| 5th | 0.79 | 0.68 | 0.92 | 0.89 | 0.76 | 1.05 |
| 10th | 0.85 | 0.73 | 0.99 | 0.95 | 0.82 | 1.11 |
| 90th | 1.21 | 1.08 | 1.36 | 1.12 | 0.99 | 1.26 |
| 95th | 1.46 | 1.30 | 1.65 | 1.11 | 0.98 | 1.26 |
| 99th | 2.26 | 1.93 | 2.65 | 1.08 | 0.88 | 1.33 |
