# Supplementary material for: Temperature extremes contribute to suicide-related help-seeking through multiple pathways: Evidence from crisis hotline data (2019–2023)
Source: PLOS Ment Health. 2026 Feb 11;3(2):e0000501. doi: 10.1371/journal.pmen.0000501 (PMC12893560; doi:10.1371/journal.pmen.0000501)
Supplement: S5 Table — (DOCX) [file pmen.0000501.s007.docx]

S5 Table. Lagged (0–2 days) generalized additive model (GAM) estimates.

| **Outcome** | **Total Events** | **Daily Mean** | **Lag 0 (Same Day)** | | | **Lag 1 (Previous Day)** | | | **Lag 2 (Two Days Prior)** | | | **Cumulative (Avg 0-2)** | | |
| --- | --- | --- | --- | --- | --- | --- | --- | --- | --- | --- | --- | --- | --- | --- |
|  |  |  | **EDF** | **p** | **% Change** | **EDF** | **p** | **% Change** | **EDF** | **p** | **% Change** | **EDF** | **p** | **% Change** |
| Mental Health | 3,127 | 0.281 | 1.01 | 0.59 | +5.6 | 1.01 | 0.55 | +6.9 | 1 | 0.69 | +3.7 | 3.14 | 0.09 | +20.9 |
| Sleep | 499 | 0.045 | 1 | 0.08 | -31.90 | 1 | 0.89 | +3.9 | 1 | 0.68 | +9.5 | 1 | 0.13 | -21.50 |
| Interpersonal | 4,617 | 0.415 | 1 | 0.59 | -3.90 | 1 | 0.99 | +0.3 | 1 | 1.00 | +0.2 | 1 | 0.40 | -3.40 |
| Basic Needs | 728 | 0.065 | 1 | 0.02 | +59.9 | 1 | 0.11 | -29.50 | 1 | 0.67 | +8.2 | 1 | 0.45 | +13.3 |
| Substance Use | 305 | 0.027 | 1.92 | 0.30 | +27.2 | 1 | 0.81 | -7.80 | 1 | 0.45 | -19.10 | 1.32 | 0.76 | -7.20 |
| Isolation | 1,242 | 0.112 | 1.01 | 0.78 | -4.40 | 1.37 | 0.81 | +7.4 | 2.64 | 0.30 | -7.60 | 1 | 0.81 | +3.5 |

**Notes:** Models adjusted for seasonality (cyclic spline), day of week, and year. Temperature percentiles: 10th = 3.9°C, 50th = 17.7°C, 90th = 24.9°C.
EDF = effective degrees of freedom for temperature smooth. ***p<0.001, **p<0.01, *p<0.05
 Change = Percent change in expected count from 10th to 90th temperature percentile
