# Supplementary material for: Temperature extremes contribute to suicide-related help-seeking through multiple pathways: Evidence from crisis hotline data (2019–2023)
Source: PLOS Ment Health. 2026 Feb 11;3(2):e0000501. doi: 10.1371/journal.pmen.0000501 (PMC12893560; doi:10.1371/journal.pmen.0000501)
Supplement: S6 Table — (DOCX) [file pmen.0000501.s008.docx]

S6 Table. Subgroup overall (0–2 days) and lagged (days 0,1,2) generalized additive model (GAM) estimates for age (≤24, ≧ 25), gender (men, women) and race (Black, white).

| **Theme** | **Lag 0** | | | **Lag 1** | | | **Lag 2** | | | **Cumulative (Lags 0–2)** | | |
| --- | --- | --- | --- | --- | --- | --- | --- | --- | --- | --- | --- | --- |
|  | **EDF** | **P** | **% change** | **EDF** | **P** | **% change** | **EDF** | **P** | **% change** | **EDF** | **P** | **% change** |
| *Youth Callers (*≤24) | | | | | | | | | | | | |
| Mental Health | 1.69 | 0.262 | -23.72 | 1 | 0.116 | 44.16 | 1 | 0.627 | 10.1 | 1.13 | 0.0982 | 21.52 |
| Basic Needs | 2.39 | 0.519 | -3.09 | 1 | 0.776 | -17.12 | 2.73 | 0.103 | 75.43 | 1.24 | 0.575 | 25.89 |
| Sleep | 1 | 0.445 | -30.62 | 1 | 0.361 | 72.79 | 1 | 0.782 | -13.05 | 1 | 0.871 | 4.69 |
| Isolation | 1.3 | 0.179 | -39.04 | 1 | 0.05 | 109.14 | 1.55 | 0.58 | -21.68 | 1.65 | 0.608 | 1.88 |
| Interpersonal | 1 | 0.701 | -5.72 | 1 | 0.303 | 21.41 | 1 | 0.536 | -9.35 | 1 | 0.654 | 3.99 |
| Substance | 1.43 | 0.522 | 129.99 | 1 | 0.688 | -36.45 | 1 | 0.765 | -24.24 | 1.21 | 0.917 | -7.94 |
| *Adult Callers (≧ 25)* | | | | | | | | | | | | |
| Mental Health | 1 | 0.271 | 28.89 | 2.32 | 0.465 | -22.71 | 1 | 0.794 | 5.83 | 2.34 | 0.575 | 6.14 |
| Basic Needs | 1 | 0.208 | 65.71 | 1 | 0.334 | -37.27 | 1 | 0.745 | -11.67 | 1 | 0.556 | -10.91 |
| Sleep | 1 | 0.421 | -30.32 | 1.22 | 0.825 | 22.91 | 1 | 0.734 | -13.66 | 1 | 0.178 | -26.24 |
| Isolation | 1 | 0.315 | 44.72 | 1 | 0.0907 | -52.67 | 1 | 0.481 | 28.22 | 1 | 0.386 | -14.49 |
| Interpersonal | 1 | 0.973 | 0.62 | 1 | 0.41 | -15.72 | 1 | 0.862 | 2.88 | 1 | 0.0938 | -13.07 |
| Substance | 1 | 0.0237 | 325.72 | 1 | 0.508 | -39.09 | 1 | 0.0348 | -69.95 | 1 | 0.317 | -27.17 |
| *Black Callers* | | | | | | | | | | | | |
| Mental Health | 1 | 0.328 | -25.56 | 1 | 0.804 | -9.61 | 4.02 | 0.013 | 84.07 | 4.66 | 0.00492 | 56.73 |
| Basic Needs | 1 | 0.343 | 77.76 | 1 | 0.0762 | -73.05 | 1 | 0.111 | 161.14 | 1 | 0.688 | 20.43 |
| Sleep | 1.29 | 0.485 | -44.68 | 1 | 0.525 | 66.78 | 1 | 0.495 | 59.14 | 1 | 0.327 | 54.99 |
| Isolation | 1.92 | 0.753 | 16.39 | 1 | 0.168 | -58.5 | 1 | 0.188 | 97.45 | 1 | 0.838 | -5.01 |
| Interpersonal | 1 | 0.692 | -8.92 | 1 | 0.71 | -11 | 1 | 0.157 | 43.5 | 1 | 0.224 | 16.52 |
| Substance | 1 | 0.539 | -43.88 | 1 | 0.15 | -80.9 | 1 | 0.0184 | 801.75 | 1 | 0.878 | -9.17 |
| *White Callers* | | | | | | | | | | | | |
| Mental Health | 1 | 0.658 | 13.12 | 1 | 0.599 | 19 | 1 | 0.269 | -25.29 | 1 | 0.99 | -0.81 |
| Basic Needs | 1 | 0.941 | -3.82 | 1 | 0.47 | 58.84 | 1 | 0.589 | -24.58 | 1 | 0.725 | 15.04 |
| Sleep | 1 | 0.989 | 0.9 | 1 | 0.0264 | -77.15 | 1 | 0.0334 | 219.17 | 1 | 0.198 | -32.65 |
| Isolation | 1 | 0.739 | 16.47 | 1 | 0.452 | -34.06 | 1 | 0.613 | 25.41 | 1 | 0.892 | -5.24 |
| Interpersonal | 1 | 0.21 | -22.57 | 1 | 0.671 | -10.26 | 1 | 0.393 | 19.48 | 1 | 0.0935 | -17.46 |
| Substance | 1 | 0.14 | 214.48 | 1 | 0.895 | -11.21 | 1 | 0.0292 | -78.54 | 1 | 0.269 | -44.14 |
| *Woman Callers* | | | | | | | | | | | | |
| Mental Health | 1 | 0.222 | -16.91 | 1 | 0.042 | 43.49 | 1 | 0.949 | -1.13 | 4.06 | 0.0851 | 25.98 |
| Basic Needs | 1 | 0.62 | 17.77 | 1 | 0.698 | -13.93 | 1 | 0.812 | 7.87 | 1 | 0.775 | 7.49 |
| Sleep | 1 | 0.409 | -24.66 | 1.68 | 0.629 | 9.68 | 2.11 | 0.279 | 8.51 | 1.21 | 0.713 | -10.2 |
| Isolation | 1 | 0.185 | -27.24 | 1 | 0.175 | 46.42 | 2.32 | 0.492 | -8.97 | 1.47 | 0.695 | 5.99 |
| Interpersonal | 1 | 0.421 | -8.83 | 1 | 0.476 | 10.65 | 1 | 0.919 | 1.22 | 1 | 0.717 | 2.45 |
| Substance | 1 | 0.432 | 55.43 | 1.02 | 0.424 | -41.83 | 1 | 0.84 | -10.35 | 1 | 0.508 | -21.9 |
| *Man Callers* | | | | | | | | | | | | |
| Mental Health | 1 | 0.583 | -9.91 | 3.28 | 0.069 | -4.61 | 1 | 1 | -0.07 | 3.39 | 0.112 | -6.81 |
| Basic Needs | 2.8 | 0.124 | 41.92 | 1.23 | 0.566 | -27.19 | 1 | 0.567 | -16.8 | 2.24 | 0.407 | -17.93 |
| Sleep | 1 | 0.026 | -59.9 | 1 | 0.559 | 34.03 | 1 | 0.999 | -0.23 | 1 | 0.061 | -43.64 |
| Isolation | 1 | 0.863 | -4.98 | 1 | 0.449 | -22.87 | 1.98 | 0.48 | -0.16 | 1 | 0.42 | -17.62 |
| Interpersonal | 1 | 0.398 | -11.75 | 1 | 0.802 | 4.64 | 2.11 | 0.567 | -5.27 | 1.76 | 0.221 | -11.81 |
| Substance | 1 | 0.0775 | 137.42 | 2.26 | 0.318 | -27.73 | 1 | 0.145 | -49.58 | 2.43 | 0.167 | -26.5 |
